# ISG15 Is Required for the Dissemination of Vaccinia Virus Extracellular Virions

**DOI:** 10.1128/spectrum.04508-22

**Published:** 2023-04-10

**Authors:** Martina Bécares, Manuel Albert, Céline Tárrega, Rocío Coloma, Michela Falqui, Emma K. Luhmann, Lilliana Radoshevich, Susana Guerra

**Affiliations:** a Department of Preventive Medicine, Public Health and Microbiology, Universidad Autónoma de Madrid, Madrid, Spain; b Department of Microbiology and Immunology, University of Iowa Carver College of Medicine, Iowa City, Iowa, USA; University of Arizona

**Keywords:** ISG15, VACV, actin tails

## Abstract

Viruses have developed many different strategies to counteract immune responses, and *Vaccinia virus* (VACV) is one of a kind in this aspect. To ensure an efficient infection, VACV undergoes a complex morphogenetic process resulting in the production of two types of infective virions: intracellular mature virus (MV) and extracellular enveloped virus (EV), whose spread depends on different dissemination mechanisms. MVs disseminate after cell lysis, whereas EVs are released or propelled in actin tails from living cells. Here, we show that ISG15 participates in the control of VACV dissemination. Infection of *Isg15^−/−^* mouse embryonic fibroblasts with VACV International Health Department-J (IHD-J) strain resulted in decreased EV production, concomitant with reduced induction of actin tails and the abolition of comet-shaped plaque formation, compared to *Isg15^+/+^* cells. Transmission electron microscopy revealed the accumulation of intracellular virus particles and a decrease in extracellular virus particles in the absence of interferon-stimulated gene 15 (ISG15), a finding consistent with altered virus egress. Immunoblot and quantitative proteomic analysis of sucrose gradient-purified virions from both genotypes reported differences in protein levels and composition of viral proteins present on virions, suggesting an ISG15-mediated control of viral proteome. Lastly, the generation of a recombinant IHD-J expressing V5-tagged ISG15 (IHD-J-ISG15) allowed us to identify several viral proteins as potential ISG15 targets, highlighting the proteins A34 and A36, which are essential for EV formation. Altogether, our results indicate that ISG15 is an important host factor in the regulation of VACV dissemination.

**IMPORTANCE** Viral infections are a constant battle between the virus and the host. While the host’s only goal is victory, the main purpose of the virus is to spread and conquer new territories at the expense of the host’s resources. Along millions of years of incessant encounters, poxviruses have developed a unique strategy consisting in the production two specialized “troops”: intracellular mature virions (MVs) and extracellular virions (EVs). MVs mediate transmission between hosts, and EVs ensure advance on the battlefield mediating the long-range dissemination. The mechanism by which the virus “decides” to shed from the primary site of infection and its significant impact in viral transmission is not yet fully established. Here, we demonstrate that this process is finely regulated by ISG15/ISGylation, an interferon-induced ubiquitin-like protein with broad antiviral activity. Studying the mechanism that viruses use during infection could result in new ways of understanding our perpetual war against disease and how we might win the next great battle.

## INTRODUCTION

Viruses are responsible for many infectious diseases, from mild illnesses such as the common cold, flu, and warts, to severe diseases such as AIDS, Ebola hemorrhagic fever, and the novel coronavirus disease 19 (COVID-19). The current severe acute respiratory syndrome-coronavirus 2 (SARS-CoV-2) pandemic is a good example of how viruses can cause global outbreaks with high mortality rates. In this sense, understanding the molecular mechanisms that operate during viral infections is essential for the development of efficient antiviral therapies against emerging viruses.

Interferon (IFN)-stimulated gene 15 (*Isg15*) encodes a small ubiquitin-like posttranslational modifier that regulates a plethora of cellular pathways through the modulation of the proteome. ISG15 exerts its functions by covalent conjugation to target proteins in a process termed ISGylation (1), or as a free molecule both inside and outside the cell (2). Intracellular unconjugated ISG15 controls the stability of target proteins through noncovalent interactions (3–5), whereas extracellular ISG15 acts as a cytokine and modulates immune cell functions by binding to lymphocyte function-associated antigen 1 (LFA-1) (6–8). ISG15 has a well-established antiviral function against diverse biomedically relevant viruses, including human immunodeficiency virus, influenza virus, SARS-CoV-2, and human herpesvirus 1 (9–12). Such antiviral activity is achieved through direct interaction of ISG15 with viral proteins or through the modulation of host proteins to limit the progression of the infection and to enhance immune responses (9). Nevertheless, viruses have developed different strategies to counteract the action of ISG15, including the cleavage of ISG15 from ISGylated proteins (11, 13), the sequestration of ISGylated viral proteins (14), or the blockage of ISGylation of target proteins (15, 16).

The *Poxviridae* is a family of large, enveloped, linear double-stranded DNA viruses that replicate entirely in the cytoplasm of infected cells. A unique feature of the *Poxviridae* family is the production of two distinct infectious forms: intracellular mature virus (MV) and extracellular enveloped virus (EV) (17, 18). MVs mediate host-to-host transmission, whereas EVs disseminate within the host causing systemic infection (19). EVs derive from MVs that bud from the plasma membrane (20), or that undergo wrapping by the *trans*-Golgi network (TGN) or endosomal membranes (21, 22). In this process, an intermediate virus form, the intracellular enveloped virus (IEV), is generated. IEVs are transported on microtubules toward the plasma membrane, which they fuse with to release EVs to the extracellular medium (23, 24); however, a variable percentage of EVs remains attached to the plasma membrane as cell-associated enveloped virus (CEV). This membrane fusion event causes IEV outer membrane-associated proteins to remain in the plasma membrane, where they induce the polymerization of actin tails to promote virion spread to neighboring cells (18). EVs are not just MV particles enclosed within a second lipid membrane, as they differ in protein composition. In *Vaccinia virus* (VACV), the proteins A25 and A26 are exclusive of MVs, whereas the proteins A36, F12, E2, B5, A34, F13, A56, and K2 are exclusive of wrapped forms (IEV and EV) (25). The production of each virus form varies among the different strains of VACV. For example, in infections with VACV Western Reserve strain (WR), MVs represent the majority of infectious progeny, whereas VACV International Health Department-J strain (IHD-J) produces a high EV/MV ratio as a result of a point mutation (K151Q) in the A34R gene, involved in EV formation and dissemination (26).

Previous research demonstrated that ISG15 has a role in the control of VACV infection (27, 28) and that VACV E3 protein counteracts the action of ISG15 (15, 27). Moreover, we previously showed that VACV reduces mitochondrial respiration of macrophages in an ISG15-dependent manner (29). Several lines of evidence argue that VACV uses an exosome-like pathway for EV formation and release (25, 30). Considering that ISG15 has been shown to impair exosome secretion by promoting the fusion of multivesicular bodies with lysosomes (31), we sought to explore whether the ISG15/ISGylation system has an impact in VACV EV formation and dissemination. Here, we show that ISG15 modulates VACV dissemination and that its absence alters the virion proteome. Infection of *Isg15^−/−^* mouse embryonic fibroblasts (MEFs) with VACV IHD-J resulted in reduced EV production, consistent with reduced actin tail formation, the accumulation of virus particles in the cytoplasm, and the abolition of comet-shaped plaques, compared to *Isg15^+/+^* MEFs. In addition, a quantitative proteomic analysis of purified virions from *Isg15^−/−^* MEFs reported an enrichment in proteins of both MVs and wrapped virions, confirming the accumulation of distinct virus forms in *Isg15^−/−^* MEFs. Lastly, the generation of a recombinant virus expressing V5-tagged ISG15 (IHD-J-ISG15) allowed us to identify viral proteins that potentially interact with ISG15, highlighting the protein A36, which is essential for the formation of actin tails (18).

In summary, our study contributes to the comprehension of how the ISG15/ISGylation system modulates VACV infection and reinforces the idea of ISG15 as an essential host factor in the coordination of viral pathogenesis and antiviral immune responses.

## RESULTS

### ISG15 is required for EV dissemination during IHD-J infection.

**FIG 1:**
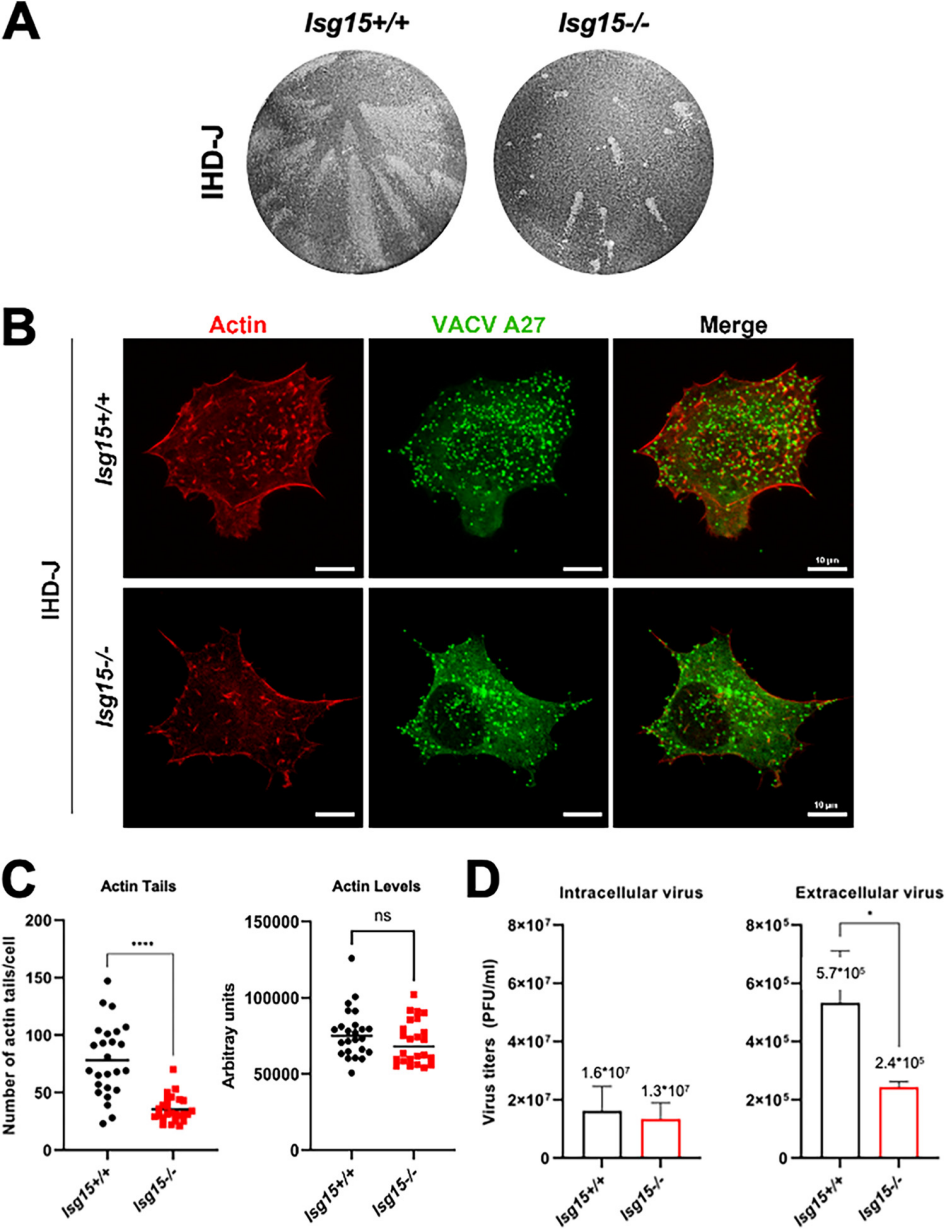
The absence of ISG15 impairs comet-shaped plaque, actin tail and EV production. (A) Comet-shaped plaque formation is dramatically reduced in *ISG15^−/−^* MEFs. Monolayers of immortalized *ISG15^+/+^* and *ISG15^−/−^* MEFs were infected with IHD-J (0.0001 PFU/cell). At 48 hpi, cells were fixed and stained with 0.2% crystal violet in 10% formaldehyde for comet-shaped plaque visualization. (B and C) Actin tail formation is significantly reduced in *ISG15^−/−^* MEFs. Immortalized *ISG15^+/+^* and *ISG15^−/−^* MEFs growing in coverslip were infected with IHD-J (2 PFU/cell). At 9 hpi, actin tails were visualized by phalloidin-staining (red), and viral particles were visualized using an anti-A27 antibody, followed by a fluorescent secondary antibody (green). Actin tails and actin levels were quantified using Leica AF or FIJI softwares (*n* = 25, each genotype in three different biologicals replicates), and actin tail numbers (means ± the SD) of individual cells are represented. The experiment has been carried out in triplicate and a representative image is shown. The specific values obtained are depicted in the graph. (D) EV release is significantly decreased in *ISG15^−/−^* MEFs. Immortalized *ISG15^+/+^* and *ISG15^−/−^* MEFs were infected with IHD-J (2 PFU/cell). At 16 hpi, infectious viral particles from supernatants (extracellular virus) and cell extracts (intracellular virus) were titrated by plaque assay. Means ± the SD from three independent experiments are represented. *, *P* < 0.05; **, *P* < 0.01; ***, *P* < 0.005; ****, *P* < 0.0001. n.s, non significative.

To analyze whether ISG15 has a role in EV release, we evaluated the formation of comet-like plaques during IHD-J infection. *In vitro*, EVs disseminate by convection, forming comet-shaped plaques consisting of a central lysis plaque (the comet “head”) and an ensemble of secondary satellite plaques (the comet “tail”) (32), allowing us to easily evaluate an enhanced production of EVs. Monolayers of *Isg15^+/+^* and *Isg15^−/−^* MEFs were infected with IHD-J (0.0001 PFU/cell) and incubated in liquid medium. At 48 h postinfection (hpi), monolayers were fixed and stained with 0.2% crystal violet in 10% formaldehyde to visualize lysis plaques. Surprisingly, a drastic reduction in the formation of comet-shaped plaques was observed in *Isg15^−/−^* MEFs (Fig. 1A), indicating that EV release is impaired in the absence of ISG15.

To study whether the reduction in comet-shaped plaques was cell line specific, we silenced *Isg15* in NIH/3T3 cells by using two specific lentiviral vectors (L1 and L2) expressing *Isg15* shRNAs. A clear reduction of *Isg15* mRNA was observed in type I IFN-treated cells transduced with both shRNAs in comparison with nontransduced cells, and with cells transduced with a control shRNA (scramble [SC]) (see Fig. S1A in the supplemental material). Western blot analysis further confirmed the interference of *Isg15* expression, since ISG15 was undetectable in L1- and L2-transduced cells (see Fig. S1B), whereas a clear induction of the protein was observed in IFN-treated control cells. *Isg15* interference in L1- and L2-transduced NIH/3T3 cells abolished the formation of comet-shaped plaques, validating our results obtained in immortalized MEFs (see Fig. S1C).

VACV relies on the cytoskeleton for dissemination. Wrapped virions are transported on microtubules to the cell surface, and CEVs induce the polymerization of actin and the formation of actin tails that propel virions to neighboring cells (17). ISG15 has been shown to modulate actin cytoskeleton dynamics (33), and several cytoskeleton proteins were identified as ISGylation targets (10). Therefore, we explored whether the absence of ISG15 alters actin tail formation during IHD-J infection. *Isg15^+/+^* and *Isg15^−/−^* MEFs were infected (2 PFU/cell, 9 h), and samples were processed for immunofluorescence analysis. Actin and MVs were labeled using Alexa Fluor 594-phalloidin and anti-VACV A27 protein-specific antibodies, respectively, and cells were imaged by confocal microscopy by triplicate. Actin tails were detected in both *Isg15^+/+^* and in *Isg15^−/−^* MEFs; although there were no differences in actin levels, a significant reduction in the number of actin tails was observed in *Isg15^−/−^* MEFs, indicating a role of ISG15/ISGylation in the modulation of actin tail formation (Fig. 1B and C). The reduction in the formation of actin tails and comet-shaped plaques was consistent with reduced extracellular virus titers in infected *Isg15^−/−^* MEFs (2 PFU/cell, 16 h, in triplicate) (Fig. 1D, right panel). Titers of intracellular virus, however, did not significantly differ between genotypes (Fig. 1D, left panel).

**FIG 2:**
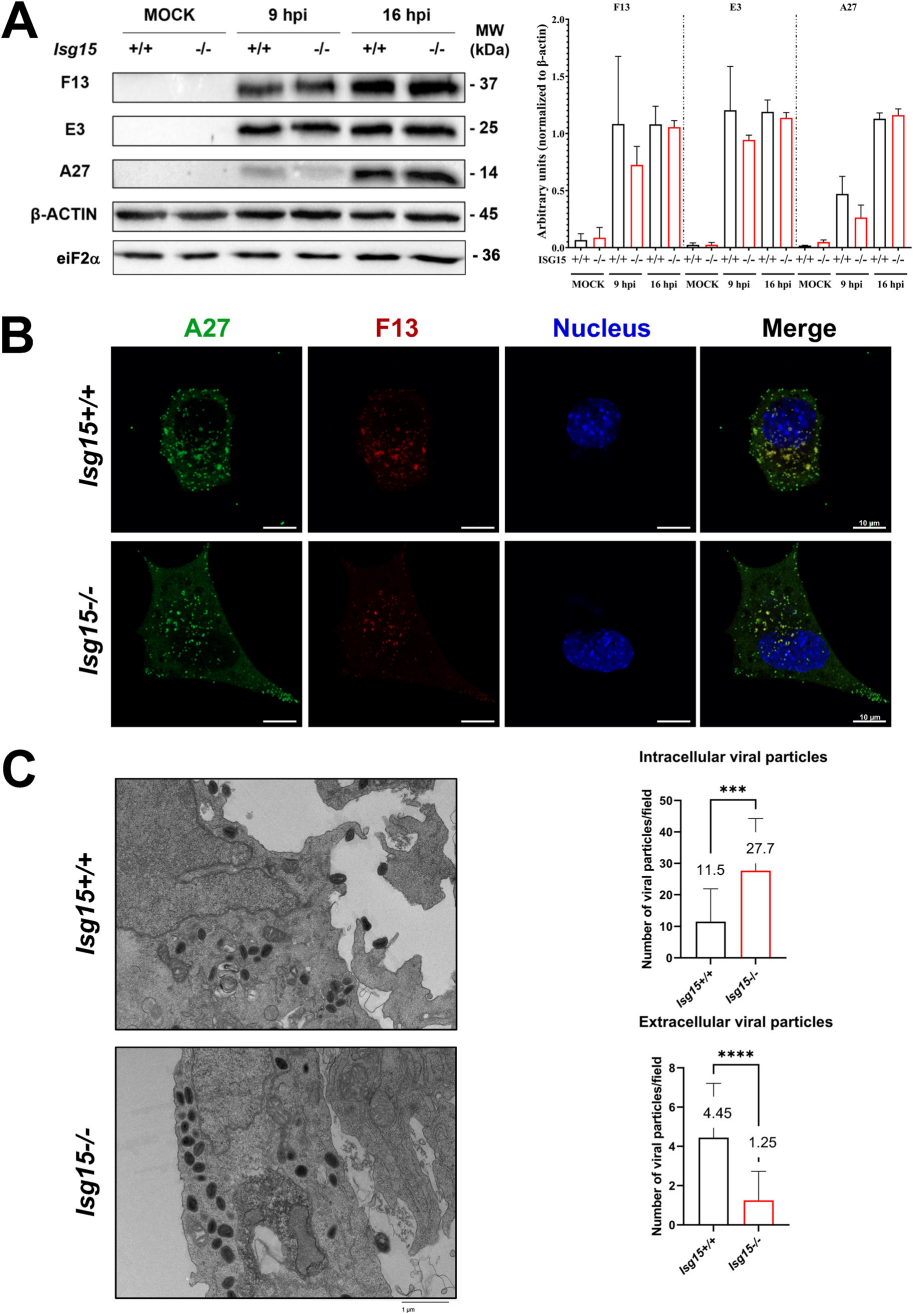
IHD-J morphogenetic process is altered in *ISG15^−/−^* MEFs. (A) Viral protein synthesis is not affected by the absence of ISG15. Immortalized *ISG15^+/+^* and *ISG15^−/−^* MEFs were infected with IHD-J (2 PFU/cell) and, at the indicated times postinfection, equal amounts of proteins from cell extracts were analyzed by Western blotting. Specific antibodies for VACV early protein E3 (*E3L*) and late proteins F13 (*F13L*) and A27 (*A27L*) were used. β-Actin and eiF2α were used as loading control. Molecular weights (MW) in kilodaltons (kDa) are indicated, based on protein standards. Protein levels of three independent experiments were analyzed with Fiji software. Means ± the SD are indicated. (B) Viral protein distribution does not change between *ISG15^+/+^* and *ISG15^−/−^* MEFs. Confocal microscopy analysis of immortalized *ISG15^+/+^* or *ISG15^−/−^* MEFs infected with IHD-J (2 PFU/cell) at 9 h postinfection. Viral proteins A27 (green) and F13 (red) were labeled by immunofluorescence; DAPI (blue) was used to stain the nuclear DNA. The experiment has been carried out in triplicate and a representative image is shown. (C) The absence of ISG15 causes accumulation of intracellular virions and a diminution in extracellular virus. Representative images of *ISG15^+/+^* and *ISG15^−/−^* MEFs infected with IHD-J (2 PFU/cell; 9 h) are shown. Intracellular or extracellular viral particles per field were quantified with Fiji software (×2,500 magnification, *n* = 20, each genotype, in three different biologicals replicates). Means ± the SD are indicated. *, *P* < 0.05; **, *P* < 0.01; ***, *P* < 0.005; ****, *P* < 0.0001. The experiment was carried out in triplicate, and a representative image is shown. The specific values obtained are depicted in the graph.

To rule out a delay in the infection of *Isg15^−/−^* MEFs as the cause of reduced actin tail formation and EV release, we analyzed the levels of early (E3) and late (A27 and F13) viral proteins by Western blotting in infected *Isg15^+/+^* and *Isg15^−/−^* MEFs (2 PFU/cell) at 9 and 16 hpi. As well, we analyzed the subcellular distribution of A27 and F13 proteins by confocal microscopy at 9 hpi. Although there was a trend of lower protein levels in infected *Isg15^−/−^* cells, neither the levels (Fig. 2A) nor the distribution (Fig. 2B) of any of the viral proteins analyzed significantly differed between *Isg15^−/−^* and *Isg15^+/+^* MEFs, indicating that the infection occurred normally in both genotypes (34).

In addition, we analyzed intracellular virus particles by transmission electron microscopy (TEM) of infected MEFs (2 PFU/cell, 9 h; by triplicate) to assess whether *Isg15^−/−^* MEFs accumulated more virus particles in the cytoplasm due to defective virus release. Interestingly, this approach revealed increased number of intracellular virus particles and a clear decrease of extracellular virus was observed of *Isg15^−/−^* MEFs (Fig. 2C). These observations were consistent with defects in EV production and dissemination in *Isg15^−/−^* MEFs and suggested that some of these intracellular viral particles might be defective, considering that the titers of intracellular infectious virus did not significantly differ between genotypes (Fig. 1D, left panel).

Lastly, to determine whether the effect of ISG15 on VACV dissemination was exclusive of IHD-J or it also affected other VACV strains, we extended our study to the WR strain. We evaluated the formation of comet-shaped plaques in WR-infected MEFs (0.0001 PFU/cell, 72 h) and observed a reduction in comet-shaped plaques in *Isg15^−/−^* MEFs (see Fig. S2A). As well, the formation of actin tails was also impaired in these cells (see Fig. S2B), indicating that the role of ISG15 in VACV dissemination is not strain specific, although EV production depends on the strain since IHD-J produces 100 times more than WR (not shown).

Altogether, our results point out the relevance of ISG15 in VACV dissemination, demonstrating that the ISG15/ISGylation system is involved in the regulation of virion egress, modulating the formation actin tails and EV release.

### Protein composition of VACV progeny is altered in the absence of ISG15.

**FIG 3:**
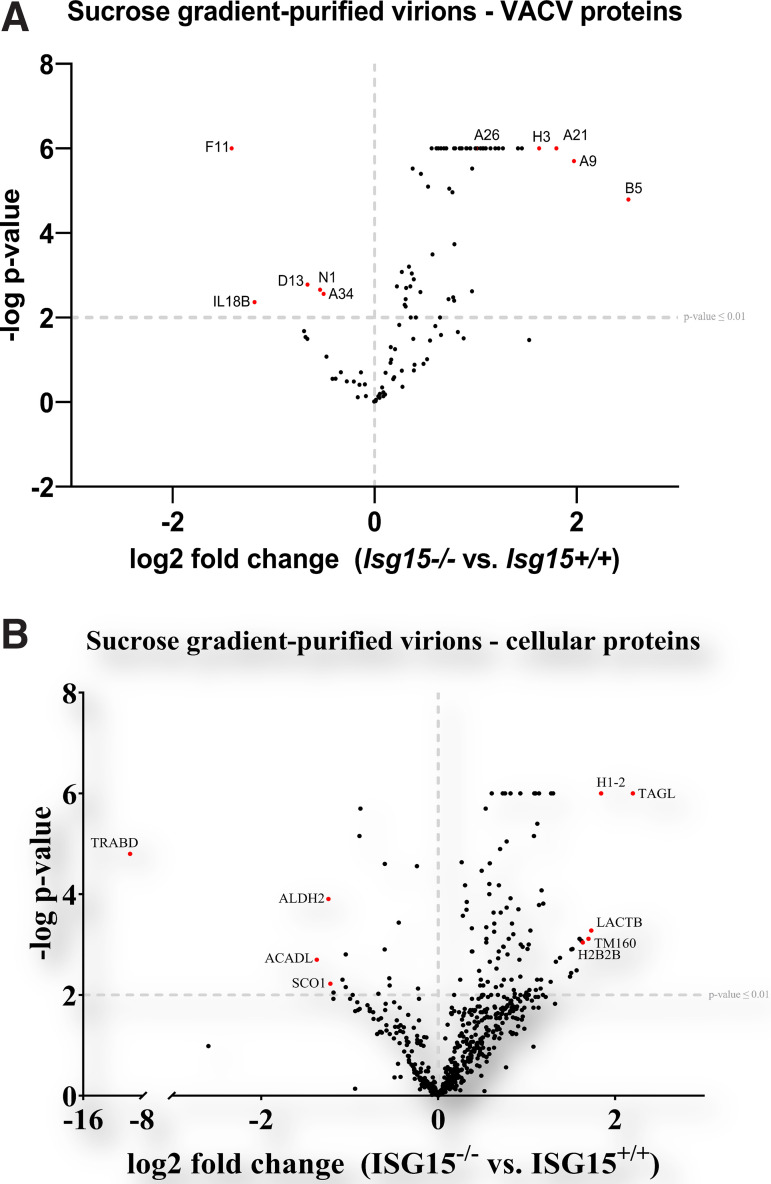
The proteomes of purified virions change in the absence of ISG15. (A) The viral protein content of highly purified virions from *ISG15^+/+^* and *ISG15^−/−^* MEFs differs between genotypes. Immortalized *ISG15^+/+^* or *ISG15^−/−^* MEFs were infected with IHD-J (0.01 PFU/cell, 48 h), and intracellular virions were purified by ultracentrifugation through a 20% sucrose cushion, followed by ultracentrifugation through a sucrose gradient (20 to 45%). Purified virions were processed for LC-MS/MS analysis. Viral proteins are represented in the volcano plot. A *P* cutoff value of ≤0.01 is indicated on the graph. Proteins of interest significantly enriched in virions isolated from *ISG15^+/+^* cells (upper left) or *ISG15^−/−^* cells (upper right) are labeled and highlighted in red. (B) The cellular protein content of highly purified virions from *ISG15^+/+^* and *ISG15^−/−^* MEFs differs between genotypes. Immortalized *ISG15^+/+^* or *ISG15^−/−^* MEFs were infected with IHD-J (0.01 PFU/cell, 48 h), and intracellular virions were purified by ultracentrifugation through a 20% sucrose cushion, followed by ultracentrifugation through a sucrose gradient (20 to 45%). Purified virions were processed for LC-MS/MS analysis. Cellular proteins are represented in the volcano plot. A *P* cutoff value of ≤0.01 is indicated on the graph. The most significantly enriched proteins in virions isolated from *ISG15^+/+^* cells (upper left) or *ISG15^−/−^* cells (upper right) are labeled and highlighted in red.

To determine whether the alterations in VACV dissemination could be caused by changes in virion proteins due to the absence of ISG15, we studied how ISG15 modulates the proteome of VACV. For that purpose, we performed a quantitative proteomic analysis with sucrose gradient-purified of similar amount of infectious MVs obtained from infected *Isg15^−/−^* and *Isg15^+/+^* MEFs. This analysis allowed us to identify and quantify numerous viral proteins whose expression could be modulated by ISG15, present in purified virions. Overall, we identified 717 proteins, 402 of which were reliably identified (present in all three biological replicates; false discovery rate [FDR] < 0.05). Of these 402 proteins, only 177 proteins were reported as significantly upregulated or downregulated (FDR < 0.01) in virions from *Isg15^−/−^* MEFs, compared to those from *Isg15^+/+^* MEFs. We found quantitative differences in the levels of 63 viral proteins comparing virions from both genotypes (Table 1). Interestingly, 58 viral proteins were significantly enriched in virions purified from *Isg15^−/−^* MEFs, whereas 5 (N1, A34, F11, D13, and IL8B) were significantly reduced (Fig. 3A).

**TABLE 1:** Viral protein signature of highly purified virions from *ISG15^+/+^* and *ISG15^−/−^* MEFs^*a*^

Protein ID	Protein	Gene	Log_2_ FC (*ISG15^−/−^* vs *ISG15^+/+^*)	–Log *P*
Q01227	Protein B5	B5R	2.51	4.79
Q85320	Virion membrane protein A9	A9L	1.97	5.70
P68712	Virion membrane protein A21	A21L	1.80	6.00
P07240	Envelope protein H3	H3L	1.63	6.00
Q89121	Serine/threonine-protein kinase 2	F10L (VPK2)	1.46	6.00
P07612	Protein L1	L1R	1.42	6.00
P12926	Core protease I7	I7L	1.27	6.00
Q76ZP7	DNA-directed RNA polymerase 133-kDa polypeptide	A24R (RP132)	1.23	6.00
P07614	Protein L3	F4 (L3)	1.19	6.00
P08583	Protein H2	H2R	1.15	6.00
P04195	Cell surface-binding protein	D8L (CAHH)	1.10	6.00
P07392	DNA-directed RNA polymerase 147-kDa polypeptide	J6R (RP147)	1.08	6.00
P16710	Virion membrane protein A16	A16L	1.07	6.00
P12927	RNA helicase NPH-II	I8R (NPH2)	1.05	6.00
P68438	RNA polymerase-associated transcription-specificity factor RAP94	H4L (RAP94)	1.02	6.00
P24758	Protein A26	A26L	1.00	6.00
P07618	Protein J5	J5L	1.00	6.00
P24361	Protein F9	F9L	0.97	5.52
P68698	DNA topoisomerase 1B	H6R (TOP1)	0.97	2.62
P05807	Nucleoside triphosphatase I	D11L (NTP1)	0.95	6.00
P21607	Protein E6	E6R	0.95	6.00
P24759	A-type inclusion protein A25	A25 (ATI)	0.91	6.00
P07616	Protein J1	J1R	0.91	6.00
P20636	Early transcription factor 82 kDa subunit	A7L (VETFL)	0.86	6.00
P16712	Transcript termination protein A18	A18R	0.86	6.00
P04300	Core protein D2	D2L	0.85	6.00
P04298	mRNA-capping enzyme catalytic subunit	D1R (MCEL)	0.84	6.00
P04308	Early transcription factor 70 kDa subunit	D6R (VETFS)	0.80	6.00
P68623	Protein L5	L5R	0.79	3.73
P24765	Protein A40	A40R	0.79	2.40
P04302	Core protein D3	D3R	0.79	6.00
P68593	Virion membrane protein A17 precursor	A17L	0.78	2.48
Q80HV7	25-kDa core protein A12	A12L	0.77	4.96
P06856	DNA polymerase	E9L (DPOL)	0.74	5.05
P68458	Protein G3	G3L	0.73	2.44
P07611	Myristoylated protein G9	G9R	0.71	6.00
P06440	Major core protein 4b	A3L (P4B)	0.71	6.00
P07617	Cap-specific mRNA (nucleoside-2′-*O*-)-methyltransferase	J3R (MCE)	0.69	6.00
P23372	Protein E8	E8R	0.66	6.00
P07239	Dual specificity protein phosphatase H1	H1L (DUSP)	0.63	6.00
P23371	Poly(A) polymerase catalytic subunit	E1L (PAP1)	0.62	6.00
P04318	mRNA-capping enzyme regulatory subunit	D12L (MCES)	0.61	6.00
P68633	Envelope protein A28	A28L	0.57	3.49
P68716	Assembly protein G7	G7L	0.57	6.00
P21603	DNA-directed RNA polymerase 30-kDa polypeptide	E4L (RP30)	0.53	5.10
P16713	Metalloendopeptidase G1	G1L	0.46	5.40
P68611	DNA-directed RNA polymerase 19-kDa subunit	A5R (RP19)	0.45	2.60
P04310	DNA-directed RNA polymerase 18-kDa subunit	D7R (RP18)	0.39	2.90
P16715	Major core protein 4a precursor	A10L (P4A)	0.38	5.52
P11258	Protein A27	A27L	0.37	3.04
P29192	Protein A6	A6L	0.35	2.74
P07242	Late transcription elongation factor H5	H5R	0.34	3.20
P03295	Core protein VP8	L4R (VP8)	0.31	2.70
P68317	DNA-directed RNA polymerase 7-kDa subunit	G5.5R (RP07)	0.31	2.44
P68638	Complement control protein C3	C3L (VCP)	0.31	2.26
P21605	Protein E3	E3L	0.30	2.30
P04021	Envelope phospholipase F13	F13L	0.27	3.08
P16714	Telomere-binding protein I1	I1L	0.22	2.74
P24761	Protein A34	A34R	−0.51	2.56
P17361	Protein N1	N1L	−0.54	2.66
P68440	Scaffold protein D13	D13L	−0.67	2.78
P17357	Interleukin-18-binding protein	IL18B	−1.19	2.36
Q80HX7	Protein F11	F11L	−1.42	6.00

a Comparison of viral proteins present in virions purified from *ISG15^−/−^* versus *ISG15^+/+^* MEFs, identified and quantified by LC-ESI-MS/MS. To identify proteins significantly enriched in each sample, a *t* test was performed (FDR = 0.05 and S_0_ = 1). Table 1 lists the protein ID (column A), the protein name, the gene name, the log_2_-fold change (FC) (*ISG15^−/−^* versus *ISG15^+/+^*) of the levels of each protein, and the statistical significance (−log *P* value). Proteins are ordered from most enriched (top) to least enriched (bottom) in *ISG15^−/−^* samples.

Among the enriched proteins in virions from *Isg15^−/−^* MEFs, we found proteins specific to wrapped virus forms, such as B5 and F13, and the protein A26, specific to MVs. These observations were consistent with defects in EV formation and the accumulation of diverse virus forms in *Isg15^−/−^* MEFs. As well, we detected a significant reduction in the levels of A34 and F11 in virions from *Isg15^−/−^* MEFs. A34 is involved in actin tail formation, EV release, the disruption of EV membranes prior to virus entry, and the localization of the proteins B5, A33, and A36 to wrapped virions (35). As well, the protein F11 promotes viral spread by modulating actin cytoskeleton through interactions with myosin 9A and RhoA (36, 37). Thus, reduced levels of A34 and F11 in virions from *Isg15^−/−^* MEFs might be related to the defects in VACV dissemination in these cells.

We also observed quantitative differences in cellular proteins comigrating with the purified virions from infected *Isg15^−/−^* and *Isg15^+/+^* MEFs. Specifically, 108 cellular proteins were reliably identified and significantly differed between samples of both genotypes (FDR < 0.01). Of the total, 15 host proteins showed lower levels in virions purified from *Isg15^−/−^* MEFs, while the rest were significantly upregulated (Table 2 and Fig. 3B). Interestingly, most of the proteins identified were related to mitochondrial metabolism and, particularly, to oxidative phosphorylation (see Fig. S3). Previously, we demonstrated that ISG15 regulates mitochondrial protein levels involved in respiration, mitochondrial dynamics, mitophagy and lipid metabolism in bone marrow-derived macrophages (29, 38), although the biological significance of their presence in samples of purified virions remains unknown.

**TABLE 2:** Cellular protein signature of highly purified virions from *ISG15^+/+^* and *ISG15^−/−^* MEFs^*a*^

Origin	Protein ID	Protein	Gene	Log_2_ FC (*ISG15^−/−^* vs *ISG15^+/+^*)	−Log *P*
Mitochondria	Q9EP89	Serine beta-lactamase-like protein LACTB, mitochondrial	*Lactb*	1.73	3.28
	Q8K2M0	39S ribosomal protein L38, mitochondrial	*Mrpl38*	1.60	3.11
	Q9D1B9	39S ribosomal protein L28, mitochondrial	*Mrpl28*	1.33	2.66
	Q8VEM8	Phosphate carrier protein, mitochondrial	*Slc25a3*	1.30	6.00
	Q64133	Amine oxidase [flavin-containing] A	*Maoa*	1.28	6.00
	P00405	Cytochrome *c* oxidase subunit 2	*Mtco2*	1.17	4.08
	Q9CPP6	NADH dehydrogenase [ubiquinone] 1 alpha subcomplex subunit 5	*Ndufa5*	1.16	2.30
	P03930	ATP synthase protein 8	*Mtatp8*	1.14	3.79
	Q9QXX4	Calcium-binding mitochondrial carrier protein Aralar2	*Slc25a13*	1.04	3.28
	Q9CQZ6	NADH dehydrogenase [ubiquinone] 1 beta subcomplex subunit 3	*Ndufb3*	1.03	2.10
	P58059	28S ribosomal protein S21, mitochondrial	*Mrps21*	1.00	2.05
	P52825	Carnitine *O*-palmitoyltransferase 2, mitochondrial	*Cpt2*	0.86	2.10
	P51881	ADP/ATP translocase 2	*Slc25a5*	0.82	6.00
	P19783	Cytochrome *c* oxidase subunit 4 isoform 1, mitochondrial	*Cox4i1*	0.78	5.05
	Q99LC5	Electron transfer flavoprotein subunit alpha, mitochondrial	*Etfa*	0.74	2.95
	P48962	ADP/ATP translocase 1	*Slc25a4*	0.73	6.00
	Q9CZW5	Mitochondrial import receptor subunit TOM70	*Tomm70*	0.71	3.63
	Q9D855	Cytochrome *b-c*_1_ complex subunit 7	*Uqcrb*	0.65	2.16
	Q9CR62	Mitochondrial 2-oxoglutarate/malate carrier protein	*Slc25a11*	0.63	3.63
	Q9DCX2	ATP synthase subunit d, mitochondrial	*Atp5pd*	0.58	4.61
	Q99JR1	Sideroflexin-1	*Sfxn1*	0.58	2.66
	Q60931	Voltage-dependent anion-selective channel protein 3	*Vdac3*	0.58	4.20
	Q62425	Cytochrome *c* oxidase subunit NDUFA4	*Ndufa4*	0.55	3.11
	Q3V3R1	Monofunctional C1-tetrahydrofolate synthase, mitochondrial	*Mthfd1l*	0.54	3.04
	Q8BFR5	Elongation factor Tu, mitochondrial	*Tufm*	0.54	3.34
	Q9CZU6	Citrate synthase, mitochondrial	*Cs*	0.54	5.70
	Q9DC69	NADH dehydrogenase [ubiquinone] 1 alpha subcomplex subunit 9, mitochondrial	*Ndufa9*	0.48	2.19
	Q9Z1P6	NADH dehydrogenase [ubiquinone] 1 alpha subcomplex subunit 7	*Ndufa7*	0.46	2.48
	Q60930	Voltage-dependent anion-selective channel protein 2	*Vdac2*	0.38	2.86
	Q9DB77	Cytochrome *b-c*_1_ complex subunit 2, mitochondrial	*U1crc2*	0.28	3.57
	Q8BMD8	Calcium-binding mitochondrial carrier protein SCaMC-1	*Slc25a24*	0.25	2.30
	Q8K2B3	Succinate dehydrogenase [ubiquinone] flavoprotein subunit, mitochondrial	*Sdha*	−0.23	2.13
	P63038	60-kDa heat shock protein, mitochondrial	*Hspd1*	−0.24	4.56
	Q06185	ATP synthase subunit e, mitochondrial	*Atp5me*	−0.45	3.44
	Q8R2Y8	Peptidyl-tRNA hydrolase 2, mitochondrial	*Ptrh2*	−0.55	2.33
	P08074	Carbonyl reductase [NADPH] 2	*Cbr2*	−0.88	5.70
	Q9CRB9	MICOS complex subunit Mic19	*Chchd3*	−0.89	5.15
	P97450	ATP synthase-coupling factor 6, mitochondrial	*Atp5pf*	−1.05	2.80
	Q5SUC9	Protein SCO1 homolog, mitochondrial	*Sco1*	−1.22	2.22
	P47738	Aldehyde dehydrogenase, mitochondrial	*Aldh2*	−1.24	3.90
	A0A0R4J083	Long-chain specific acyl-CoA dehydrogenase, mitochondrial	*Acadl*	−1.37	2.70
Nucleus	P15864	Histone H1.2	*H1-2*	1.84	6.00
	Q64525	Histone H2B type 2-B	*Hist2h2bb*	1.64	3.04
	P43277	Histone H1.3	*H1-3*	1.49	2.36
	P17095	High mobility group protein HMG-I/HMG-Y	*Hmga1*	1.38	2.74
	P62806	Histone H4	*H4c1*	1.10	6.00
	P43274	Histone H1.4	*H1-4*	1.09	6.00
	Q61656	Probable ATP-dependent RNA helicase DDX5	*Ddx5*	1.02	3.00
	P43275	Histone H1.1	*H1-1*	0.93	3.00
	P43276	Histone H1.5	*H1-5*	0.93	6.00
	Q8CGP1	Histone H2B type 1-K	*H2bc12*	0.93	2.36
	P15864	Histone H1.2	*H1-2*	0.91	3.70
	C0HKE1	Histone H2A type 1-B	*H2ac4*	0.85	3.20
	P27661	Histone H2AX	*H2ax*	0.78	2.80
	P97315	Cysteine and glycine-rich protein 1	*Csrp1*	0.72	2.19
	P10922	Histone H1.0	*H1-0*	0.69	2.86
	P11031	Activated RNA polymerase II transcriptional coactivator p15	*Sub1*	0.68	2.73
	P62827	GTP-binding nuclear protein Ran	*Ran*	0.66	2.52
	P14602	Heat shock protein beta-1	*Hspb1*	−1.08	2.30
Cytoplasm	Q7TT52-2	Isoform 2 of protein phosphatase 1 regulatory subunit 14D	*Ppp1r14d*	1.57	2.49
	P41105	60S ribosomal protein L28	*Rpl28*	1.51	2.90
	P86048	60S ribosomal protein L10-like	*Rpl10l*	1.16	2.13
	Q8BK63	Casein kinase I isoform alpha	*Csnk1a1*	1.12	2.30
	O08528	Hexokinase-2	*Hk2*	1.01	2.91
	P62830	60S ribosomal protein L23	*Rpl23*	0.96	2.95
	Q9D8E6	60S ribosomal protein L4	*Rpl4*	0.83	2.30
	Q9QYI3	DnaJ homolog subfamily C member 7	*Dnajc7*	0.80	3.11
	P50543	Protein S100-A11	*S100a11*	0.69	4.18
	P60710	Actin, cytoplasmic 1	*Actb*	0.61	6.00
	P10126	Elongation factor 1-alpha 1	*Eef1a1*	0.49	4.46
	P58252	Elongation factor 2	*Eef2*	0.38	3.32
	Q9DBJ1	Phosphoglycerate mutase 1	*Pgam1*	−0.55	2.19
	P17751	Triosephosphate isomerase	*Tpi1*	−0.61	2.90
Cytoskeleton	P62737	Actin, aortic smooth muscle	*Acta2*	1.19	2.00
	Q91VH6	Protein MEMO1	*Memo1*	1.12	5.40
	Q08093	Calponin-2	*Cnn2*	0.84	3.41
	Q8BMK4	Cytoskeleton-associated protein 4	*Ckap4*	0.76	6.00
	Q64727	Vinculin	*Vcl*	0.56	2.26
	P05213	Tubulin alpha-1B chain	*Tuba1b*	0.39	2.86
	E9Q616	AHNAK nucleoprotein (desmoyokin)	*Ahnak*	−0.60	4.60
	P13020	Gelsolin	*Gsn*	−1.05	2.16
Endosome/Lysosome	P35278	Ras-related protein Rab-5C	*Rab5c*	1.08	5.15
	Q9D1G1	Ras-related protein Rab-1B	*Rab1b*	0.77	3.73
	P53994	Ras-related protein Rab-2A	*Rab2a*	0.74	2.96
	P51150	Ras-related protein Rab-7a	*Rab7a*	0.70	4.89
	P62821	Ras-related protein Rab-1A	*Rab1A*	0.64	3.41
	P46638	Ras-related protein Rab-11B	*Rab11b*	0.63	3.26
	Q9CQD1	Ras-related protein Rab-5A	*Rab5a*	0.51	2.19
Endoplasmic reticulum	Q5SYH2	Transmembrane protein 199	*Tmem199*	1.62	3.07
	Q8BSY0	Aspartyl/asparaginyl beta-hydroxylase	*Asph*	1.50	2.44
	Q9CQS8	Protein transport protein Sec61 subunit beta	*Sec61b*	1.27	2.26
	Q922Q8	Leucine-rich repeat-containing protein 59	*Lrrc59*	0.82	3.92
	Q91YQ5	Dolichyl-diphospho-oligosaccharide/protein glycosyltransferase subunit 1	*Rpn1*	0.74	2.30
	P08113	Endoplasmin	*Hsp90b1*	0.48	2.30
Other	P37804	Transgelin	*Tagln*	2.20	6.00
	Q9D938	Transmembrane protein 160	*Tmem160*	1.70	3.11
	Q9CQ76	Nephrocan	*Nepn*	1.52	2.91
	Q62523	Zyxin	*Zyx*	1.19	3.81
	P61205	ADP-ribosylation factor 3	*Arf3*	1.11	2.10
	P11499	Heat shock protein HSP 90-beta	*Hsp90ab1*	0.92	2.70
	Q9WVA4	Transgelin-2	*Tagln2*	0.58	4.00
	O35639	Annexin A3	*Anxa3*	0.32	3.85
	P07356	Annexin A2	*Anxa2*	0.32	3.69
	P67778	Prohibitin	*Phb*	0.31	4.18
	P63017	Heat shock cognate 71-kDa protein	*Hspa8*	0.27	4.63
	P20152	Vimentin	*Vim*	−1.18	2.05
	Q99JY4	TraB domain-containing protein	*Trabd*	−9.62	4.80

a Comparison of cellular proteins present in virions purified from *ISG15^−/−^* versus *ISG15^+/+^* MEFs, identified and quantified by LC-ESI-MS/MS. To identify proteins significantly enriched in each sample, a *t* test was performed (FDR = 0.05 and S_0_ = 1). Table 2 lists the protein subcellular location (referred as “origin”), the protein ID, the protein name, the gene name, the log_2_-fold change (FC) (*ISG15^−/−^* versus *ISG15^+/+^*) of the levels of each protein, and the statistical significance (−log *P* value). Proteins are ordered from the most enriched (top) to the least enriched (bottom) in *ISG15^−/−^* samples within each “protein origin” group.

### ISG15 binds VACV proteins.

**FIG 4:**
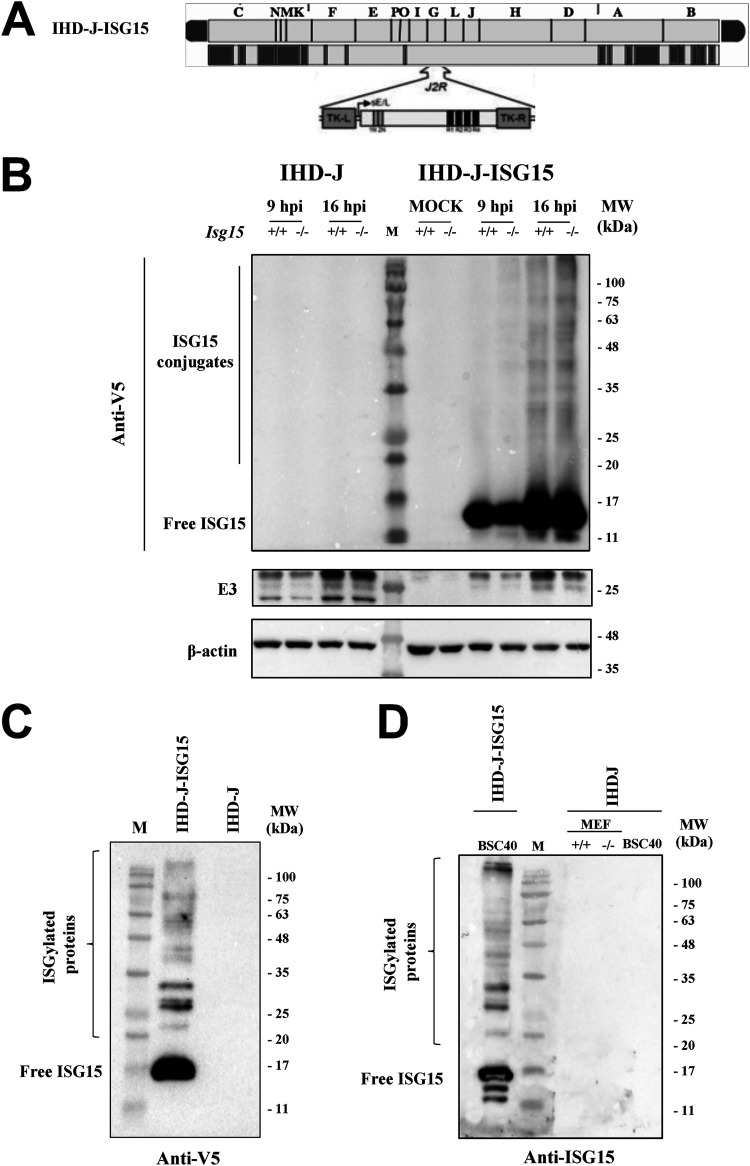
Generation and *in vitro* characterization of IHD-J-ISG15. (A) Scheme of the IHD-J-ISG15 genome map. The full-length murine ISG15 with a V5 epitope fused to its N terminus was inserted within the VACV TK viral locus (*J2R*), and its expression was driven by the VACV E/L promoter (B) Expression of V5-ISG15 fusion protein. Immortalized *ISG15^+/+^* or *ISG15^−/−^* MEFs were mock infected or infected at 2 PFU/cell with IHD-J or IHD-J-ISG15. At the indicated times postinfection, proteins were separated by SDS-PAGE, and the expression of viral early (E3) protein was analyzed by Western blotting with specific antibodies. Actin was used as a loading control. (C and D) Purified IHD-J-ISG15 virions contain ISGylated proteins and free ISG15. Purified IHD-J or IHD-J-ISG15 from BSC40 cells or immortalized *ISG15^+/+^* and *ISG15^−/−^* MEFs were fractionated by SDS-PAGE, and the expression of V5-ISG15 (C) and ISG15 (D) was analyzed by Western blotting with specific antibodies. Molecular weights (MW) in kilodaltons (kDa) are indicated based on protein standards (M).

ISGylation, unlike other posttranslational modifications that happen in nature, occurs at very low levels, and although it has a notable effect, it is very difficult to appreciate with current technologies (39). These difficulties regarding the identification of ISGylated peptides motivated us to develop a recombinant virus expressing murine ISG15 (IHD-J-ISG15) to assess whether ectopically expressed ISG15 binds VACV proteins when coexpressed. To distinguish endogenous ISG15 from that expressed by the recombinant virus, the latter was tagged with a V5 epitope at its N terminus. The V5-ISG15 construct was inserted into the VACV thymidine kinase (TK) locus of parental IHD-J, under the transcriptional control of the VACV early/late promoter. The IHD-J-ISG15 virus was generated following an infection-transfection protocol (40), and its genome is depicted in Fig. 4A. The correct insertion and purity of recombinant IHD-J-ISG15 viruses were analyzed by PCR using primers annealing in the VACV TK-flanking regions that confirmed the presence of the full-length murine ISG15 gene. Moreover, the correct sequence of full-length ISG15 gene inserted in the VACV TK locus was also confirmed by DNA sequencing (not shown).

To demonstrate that IHD-J-ISG15 constitutively expressed the full-length murine ISG15, *Isg15^+/+^* and *Isg15^−/−^* MEFs were mock infected or infected with 0.5 PFU/cell of parental IHD-J or IHD-J-ISG15 for 9 and 16 h, and the expression of recombinant ISG15 was analyzed by Western blotting with an anti-V5 specific antibody. The results show that both monomeric ISG15 and ISGylated proteins were detected during IHD-J-ISG15 infection (Fig. 4B). Next, to determine whether virus-expressed ISG15 could be conjugated to virion proteins, sucrose gradient-purified IHD-J and IHDJ-ISG15 virions were analyzed by Western blotting. Using a V5-specific antibody, we detected protein ISGylation in protein extracts from purified intracellular IHDJ-ISG15 virions, as expected (Fig. 4C). Using an ISG15 antibody, we analyzed ISG15 levels in IHDJ-ISG15 MVs purified from BSC40 cells and in IHD-J MVs purified from BSC40 cells and *Isg15^+/+^* and *Isg15^−/−^* MEFs (Fig. 4D). We only detected a clear signal in the recombinant virions, indicating that the absence of ISG15/ISGylation signal in IHD-J virions is probably due to the low levels of ISGylation produced, which makes it undetectable by Western blotting (Fig. 4C).

**FIG 5:**
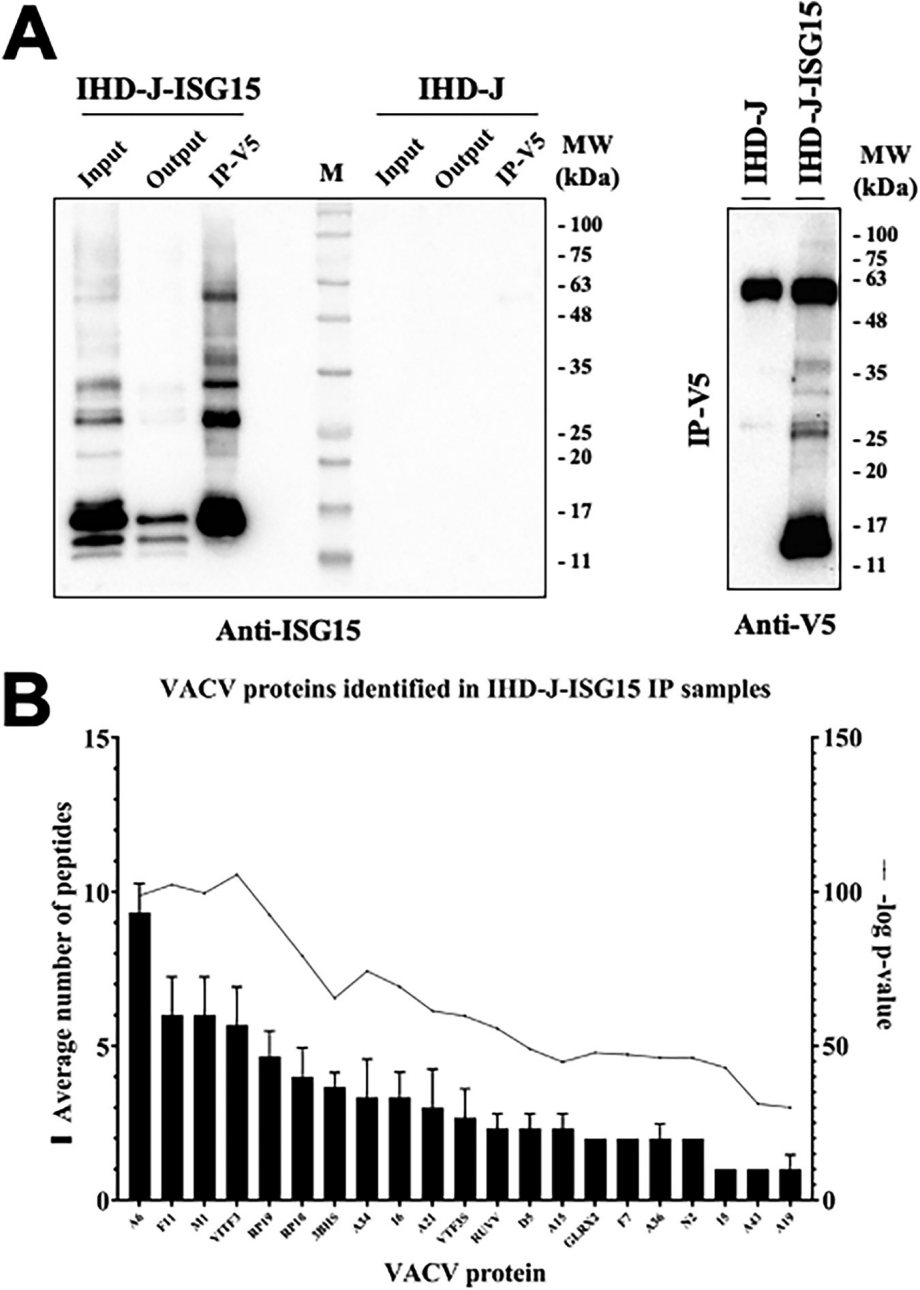
Several VACV proteins interact with ISG15. (A) Immunoprecipitation of ISG15 and ISGylated proteins from IHD-J-ISG15 virions. Sucrose gradient-purified IHD-J-ISG15 and IHD-J virions were processed, and V5-ISG15-conjugated viral proteins were subjected to immunoprecipitation with a V5 antibody. (Left panel) The total protein extract before (Input) and after (Output) the immunoprecipitation assay and the immunoprecipitated proteins (IP-V5) were fractionated by SDS-PAGE, and ISG15 and ISGylated proteins were analyzed by Western blotting with an ISG15-specific antibody. (Right panel) The immunoprecipitated proteins were analyzed by Western blotting with an anti-V5-specific antibody. Purified IHD-J virions were used as control. Molecular weights (MW) in kDa are indicated based on protein standards (M). (B) Identification of VACV proteins as potential targets of ISGylation. Immunoprecipitated protein extracts from sucrose gradient-purified IHD-J-ISG15 and IHD-J virions were analyzed by LC-ESI-MS/MS to identify VACV proteins that interact with ISG15. The graph collects 21 proteins that were found exclusively in IHD-J-ISG15 immunoprecipitated samples, identified as potential ISG15 interactors. The average numbers of peptides and –log *P* values from three biological replicates are represented for each protein.

To identify ISGylated viral proteins, we performed immunoprecipitation assays with IDH-J and IHD-J-ISG15 sucrose gradient-purified MVs to capture and detect V5-ISG15 targets, using a V5 antibody. Western blot analysis of the immunoprecipitated extracts showed that several proteins reacted against an ISG15-specific antibody (Fig. 5A). To identify these proteins, the samples were subjected to liquid chromatography with electrospray ionization and tandem mass spectrometry (LC-ESI-MS/MS). A high-resolution short gradient was performed, and 21 VACV proteins were identified in the immunoprecipitated extract (Table 3 and Fig. 5B). Several of the identified viral proteins are involved in the regulation of cytoskeleton dynamics (F11, A34, and A36), virion morphogenesis (A6, A15, and A19), and virion entry (A21). Our results clearly demonstrated that IHD-J-ISG15 a very efficient tool to detect ISG15-interacting proteins.

**TABLE 3:** Immunoprecipitated viral proteins from purified IHD-J-ISG15 virions^*a*^

Protein ID	Protein	Gene	Function	Abundance (avg peptide no.)	−Log *P*
P29192	Protein A6	A6L	Essential in IV-MV transition	9.33	98.76
Q80HX7	Protein F11	F11L	Microtubule dynamics; cell-to-cell virus spread	6	102.34
P20640	Ankyrin repeat protein M1	M1L	Resistance to α-amanitin	6	99.6
Q80HV2	Intermediate transcription factor 3 large subunit	A23R (VITF3L)	Transcription of intermediate genes	5.67	105.63
P68611	DNA-directed RNA polymerase 19-kDa subunit	A5R (RP19)	Transcription of early genes; associates with ETF1	4.67	92.51
P04310	DNA-directed RNA polymerase 18-kDa subunit	D7R (RP18)	Transcription of early genes; associates with ETF1	4	79.22
P21097	3β-Hydroxysteroid dehydrogenase/delta 5→4-isomerase	A44L (3BHS)	Production of steroids; modulate inflammatory response	3.67	65.45
P24761	Protein A34	A34R	EV formation and egress	3.33	74.31
P68462	Telomere-binding protein I6	I6L	Binds to viral telomeric sequence; genome encapsidation	3.33	69.19
P20996	Virion membrane protein A21	A21L	Virus entry; fusion of virus and host membranes	3	61.42
P68720	Intermediate transcription factor 3 small subunit	A8R (VTF3S)	Transcription of intermediate genes	2.67	59.69
Q80HV3	Resolvase A22	A22R (RUVV)	DNA replication: cleavage of DNA concatamers to form viral genomes	2.33	55.64
P21010	Primase D5	D5R	Initiation of DNA replication	2.33	49.04
P20992	Core protein A15	A15L	Morphogenetic complex; formation of virosomes	2.33	44.83
P68461	Glutaredoxin-2	G4L (GLRX2)	Virion morphogenesis and viral replication; formation of disulfide bonds in intracellular virion membrane	2	47.85
P21016	Protein F7	F7L		2	47.18
P68619	Protein A36	A36R	Intracellular transport of virions to the host cell surface; actin tail formation	2	46.14
P14357	Protein N2	N2L	Inhibition of host’s immune response; inhibition of IRF3 signaling	2	46.03
P12924	Protein I5	I5L	Enhancement of viral replication and virulence	1	42.96
P21065	Protein A43	A43R	Enhancement of intradermal lesion formation	1	31.23
P68714	Protein A19	A19L	Maturation of immature virions	1	30.03

a Extracts from IHD-J-V5-ISG15 were immunoprecipitated using an V5 specific antibody and analyzed by LC-ESI-MS/MS. Table 3 lists the protein ID, the protein name, the gene name, the protein function, the abundance (expressed as average peptides detected for each protein in the proteomic analysis of three biological replicates per condition), the statistical significance (–log *P*). Proteins are ordered by abundance. Protein extracts of purified IHD-J virions were immunoprecipitated as negative controls.

Altogether, these results indicate that ISG15 modulates the VACV proteome, which is expected to impact VACV infection at different levels.

## DISCUSSION

Poxviruses are the only known viruses that produce more than one infectious form during their infectious cycle. The distinct infective particles differ not only antigenically and structurally but also in the way that they exit the cell (17, 41, 42). While MVs are released from the infected cell after cell lysis, the EVs bud directly from the plasma membrane without necessarily encouraging cellular death. Although *Variola virus* (VARV; the causal agent of smallpox) has been eradicated, poxviruses are currently back in the news after the recent outbreak of monkeypox virus (MPXV), a zoonotic orthopoxvirus that also causes disease in humans, although it results in notably lower mortality rates compared to VARV (43). The possibility that, in the future, MPXV conquers the ecological niche once occupied by VARV exists; therefore, it is important to understand the host restriction factors that control the different poxvirus dissemination mechanisms.

ISG15 is one of the most highly expressed genes in response to IFN and encodes a protein involved in a ubiquitin-like posttranslational modification denominated ISGylation (9). We used the WR and IHD-J strains of VACV and, although we demonstrated that ISG15 is essential for the correct dissemination in both strains, the higher production of EVs by IHD-J (44, 45) made us focus our work on this strain. In contrast to what we observed with exosomes (31), our results clearly show that the absence of ISG15 is associated with a reduction in EV production and comet-shaped plaques, indicating that the mechanism by which ISG15 may be regulating both processes, EV and exosomes, might differ. This does not mean that they do not share similarities; specifically, both processes are blocked by IFN treatment (46). Even though IFN substantially increases ISG15 and ISGylation, we cannot rule out that this effect is caused by any other IFN-stimulated pathway.

Although there is a clear defect in EV release in *Isg15^−/−^* cells, the production of viral proteins did not differ between *Isg15^+/+^* and *Isg15^−/−^* cells, as observed with A27, the classical marker for MV/EV, and F13, the major envelope protein of EV, that associates with Golgi membranes and is essential for EV formation (17). These results suggest that the absence of ISG15 does not affect the correct kinetics of viral gene expression (34). The interaction of F13 with the MV surface is a requirement for the association between MVs and wrapping membranes (47), and its absence leads to a dramatic reduction in viral spread and plaque size (48). As mentioned above, we did not observe any differences in the levels of F13 between genotypes, although we cannot rule out that the absence of ISG15 affects the proper function of F13 in this process. IHD-J is very efficient in TGN-mediated virion wrapping and the release of extracellular enveloped virus (45); however, as seen by TEM, intracellular virus particles were increased in IHD-J-infected *Isg15^−/−^* cells. Surprisingly, when we quantified the intracellular infectious virions by plaque assay, we did not observe any difference between genotypes, suggesting that perhaps many of the accumulated particles in *Isg15^−/−^* cells might be defective and therefore not infectious. In addition, since our data indicate a decrease in infectious EVs in infected *Isg15^−/−^* cells, we hypothesize that the ISG15/ISGylation system plays a regulatory role during virion egress.

This irregular viral spread observed in *Isg15^−/−^* MEFs is also associated with a quantitative difference in the protein signature of IHD-J virions (Tables 1 and 2). The quantitative proteomic analysis of gradient-purified intracellular virus showed a general increase of cellular and viral proteins in virions grown in *Isg15^−/−^* MEFs compared to those grown in *Isg15^+/+^* MEFs (Table 1). The fact that many mitochondrial proteins were present in purified virions might indicate that these proteins were copurified with the virions. The mitochondrial proteins detected are mainly involved in oxidative phosphorylation, and their levels were higher in purified from *Isg15^−/−^* MEFs (Table 2). We have previously shown that ISG15 is necessary for proper oxidative phosphorylation and that the absence of ISG15 results in the accumulation of mitochondrial proteins (29). However, given the large size of VACV and its complexity, it is possible that many of these proteins were engulfed within the virions during the morphogenetic process. Interestingly, in a preliminary proteomic study of virion-enriched cell extracts (data not shown), we found the Ring Finger Protein 213 (RNF213) among the less abundant cellular proteins in *Isg15^−/−^* samples. This protein was described as a sensor of ISGylated proteins, which showed antimicrobial activity against bacteria and viruses *in vitro* and *in vivo* (49). This observation suggests that the interaction between ISG15 and RNF213 might have a relevant role in the antiviral response against VACV.

Focusing on viral proteins present in both types of virions, we identified 58 proteins that were significantly upregulated and only 5 that were significantly reduced (N1, A34, F11, D13, and IL8B) in virions purified from *Isg15^−/−^* MEFs compared to those from *Isg15^+/+^* MEFs (Table 1). Our results are in line with previous proteomic analyses of purified virions (50–52). Interestingly, we identified several proteins from EVs, which levels were upregulated in intracellular virions purified from *Isg15^−/−^* MEFs, suggesting that a part of the EV virus has been retained inside the cell in the absence of ISG15. The protein B5 was the most upregulated protein in virions purified from *Isg15^−/−^* cells. B5 has been shown to be involved in IEV formation and actin tail polymerization (53). A33, A34, and B5 are critical for the efficient production of infectious EV, and interactions among these proteins are important for their localization and incorporation into the outer extracellular virion membrane (54). Our proteomic analysis reported a reduction in the levels of A34 in virions purified from *Isg15^−/−^* MEFs. The absence of A34 was linked to a strong decrease in the amounts of B5 present in EV (54); however, little is known about how a dysregulation in A34 levels, other than its complete absence, affects B5. Furthermore, the presence of B5 at the surface of the EV is required for actin tail formation after contact with the membrane of cells expressing A33 and A36 (55). Our results indicate that, in the absence of ISG15, the release of EV and the formation of actin tails are impaired. Moreover, in our study, we detected increased levels of B5 in virions purified from *Isg15^−/−^* MEFs. We hypothesize that the absence of ISG15 causes alterations in virion egress, which leads to an accumulation of B5-containing viral progeny. This would explain the higher levels of B5 in purified virions from *Isg15^−/−^* MEFs and a reduction in actin tail formation, since these B5-containing virions would not be released and therefore could not induce actin polymerization at the cell surface.

The protein F11, involved in EV formation and virus spread (37), was identified as the most downregulated protein in virions from *Isg15^−/−^* MEFs. F11 modulates the cortical actin cytoskeleton and enhances the release of virions (36); therefore, a reduction in the levels of F11 is consistent with our hypothesis of impaired virion egress and our observations of decreased EV release. Interestingly, we identified F11 and A34 as proteins that interact with ISG15 (Table 3). Considering that ISG15 regulates host and pathogen protein dynamics, it is possible that its absence alters the localization and interaction of viral proteins involved in virion maturation, actin tail formation, and EV formation (10, 33).

A26 was one of the viral proteins increased in virions grown in the absence of ISG15, as indicated by the proteomic analysis of purified virions and a previous proteomic analysis with virion-enriched cell extracts. Moreover, an increase in A26 levels was detected by Western blot analysis of infected *Isg15^−/−^* MEFs (not shown). The A26 protein is present in MVs but absent in wrapped virions (56); therefore, the accumulation of intracellular viral particles reported in *Isg15^−/−^* MEFs could explain these observations. Furthermore, such accumulation could also explain the increased levels of A26 in purified virions from *Isg15^−/−^* MEFs, as the content of MVs is expected to be higher than in virions purified from *Isg15^+/+^* MEFs. In some *Orthopoxvirus* species, A26 participates in the attachment of MVs to the cell surface and the incorporation of MVs into A-type inclusions (ATIs) (57). Those structures provide stability and protection to MVs ensuring a proper transmission between surrounding cells, and the presence of the entire AT1 protein (A25) is required for its formation (57). In VACV, the AT1 protein is truncated and does not aggregate into ATIs; however, it still associates with and retains A26 (58). Several studies suggest that A26 mediates in the decision between virion wrapping and the formation of A-type inclusions, suggesting that A26 acts as a switch to enhance the production of MVs at the expense of EVs (56, 59). A26 also enhances retrograde transport of MVs (58) and participates in cell attachment by interacting with laminin (60). Recently, Holley et al., in line with our studies, demonstrated that A26 expression downregulates EV production, pointing out that MV maturation is controlled by the abundance of A26 (59).

Our work shed light on a novel mechanism in which ISG15 is a host factor for the control of EV production, in line with an accumulation of A26, and higher levels of relevant proteins for VACV, such as B5, and F13, in virions purified from *Isg15^−/−^* MEFs. Therefore, we wondered whether ISG15 regulates these proteins through ISGylation. It has been shown that, during viral infections, the adequate regulation of posttranslational modifications (PTMs) on the same or nearby residues of the same protein, is important to perpetuate the infection (12). Thus, it is possible that the ISGylation of VACV proteins alters other PTMs required for their proper function. Our previous efforts to immunoprecipitate ISG15 and ISGylated proteins from purified virions were unsuccessful, so we decided to generate a recombinant virus expressing V5-tagged ISG15. ISG15 is conjugated to *de novo*-synthesized proteins in a cotranslational manner, what facilitates ISGylation of viral proteins during infection (61). However, replicative VACV strains downregulate ISG15 expression and shut off translation of host proteins (62). Considering that ISGylation occurs to *de novo*-synthesized proteins, the detection of ISGylated proteins after VACV infection is very difficult, especially at late stages of infection. To overcome this drawback, the expression of recombinant ISG15 was controlled by an early/late viral promoter that allows the expression of ISG15 throughout the viral cycle. Thus, ISG15 was synthesized at the same time as viral proteins, which increased the efficiency in viral protein ISGylation. Immunoprecipitation of V5-tagged ISG15 from IHD-J-ISG15 virion protein extracts and the subsequent proteomic analysis identified 21 VACV proteins capable of interacting with ISG15. However, when we used the anti-ISG15 antibody to immunoprecipitate ISG15, we did not detect any protein (not shown), indicating that we can only identify ISGylated proteins in purified virions that overexpressed ISG15. This suggests that ISGylation with endogenous ISG15 occurs at very low levels, making it difficult to detect with current technologies (39). Furthermore, the identification of endogenous ISG15 substrates through immunoprecipitation remains suboptimal due to the inefficiency of ISG15 antibodies in targeting ISGylated proteins (39).

Among the detected VACV proteins, we identified envelope proteins (e.g., A36, A34, and F11), core proteins (e.g., A15, A19, A6, and A21), proteins with enzymatic activity (e.g., F7, GLRX2, and 3BHS), proteins involved in replication and transcription of the viral genome (e.g., VITF3, VTF3S, RP18, RP19, and RUVV), and proteins involved in the antiviral response (such as N2) (63).

The protein A36 is predominantly associated with the outer IEV envelope and, after virion release, it accumulates in plasma membrane beneath the CEV, where it is necessary for actin tail formation (64). It was demonstrated that multiple interactions between IEV membrane proteins exist, which are relevant for IEV assembly and actin tail formation (17, 65). An efficient virus release required a tight tethering to the host cell through interactions mediated by viral envelope proteins (66). Since the proteins A34 and A36 participate in actin tail formation, the fact that they appear as potential ISG15 targets suggests that an impairment of the interplay between ISG15 and these proteins in *Isg15^−/−^* cells could also contribute to the reduction in actin tails. In future work, we will try to elucidate the biological significance of this interaction (perhaps ISGylation) in the context of VACV infection. Unfortunately, the A26 protein was not present among our immunoprecipitated candidates, indicating that the interaction between ISG15 and A26 is unlikely to occur.

Although all our work has focused on the role of ISG15 in the regulation of the cytoskeleton in late events of the viral cycle, such as actin tail production, it is important to note that VACV also depends on the cytoskeleton to enter the cell by macropinocytosis, which is highly dependent on actin dynamics at the cell surface (67). Although our data do not show any statistically significant differences in the expression of viral proteins over time, future studies will be performed to analyze whether ISG15 may also be involved in viral entry.

The major finding of the present manuscript is that ISG15 and ISGylation regulates VACV dissemination; however, this should not be interpreted as a proviral effect of ISG15 during VACV infection, as it has been described for other viruses, such as hepatitis C virus or hepatitis B virus (reviewed in reference 9). Even though previously we thought of ISG15 exclusively as an antiviral protein (15), as a recent review has pointed out (68), ISG15 roles are much more complex that we originally thought, being a crucial piece in the control of the innate immune response (68). The role of ISG15 in EV production depends on the viral strain analyzed, which led us to choose IHD-J to appreciate differences in EV production since other strains, such as WR, do not allow it. We hypothesize that the fact that ISG15 favors the production of EVs might facilitate the early detection of these particles by the immune system and the rapid stimulation of immune responses, considering that EVs are susceptible to antibody-mediated neutralization (69–71). We believe that ISGylation regulates the polymerization of actin tails in a highly regulated manner and, given the relevance of ISGylation for many cell functions, viruses exhibit mechanisms to control ISGylation levels. In the case of VACV, it seems to be mediated by the protein E3 (15, 27). Although our hypothesis is entirely speculative, similar mechanisms have been observed during bacterial infections. It has been shown that the production of bacterium-derived extracellular vesicles is a vehicle for spreading of pathogen-associated molecular patterns, which stimulate the host’s innate immune response through the activation of pattern-recognition receptors, stimulator of interferon genes (STING), and inflammasome signaling pathways (72).

Many questions remain open to the fact that poxviruses produce two different infectious forms with different characteristics. Is this advantageous, and, if so, why is the percentage of EVs produced so low compared to the MVs? Is it more advantageous to produce MVs in large quantities and wait for the cell to lyse or does it make sense to release few virions into the extracellular medium as soon as possible? Regarding the pathogenicity, it has been reported that EV allows dissemination across long distances to infect distant tissues within the host (41). IHD-J-infected mice showed reduced mortality compared to WR-infected mice (44), suggesting that higher EV production and dissemination within the host does not significantly influence virulence; the WR strain however, is highly pathogenic despite its low EV production (54). Regarding VARV, the increased comet formation phenotypes in several strains and the subsequent EV production is associated with geographical and phylogenetic origin but not with increased pathogenicity (73).

In summary, our results suggest that ISG15 is a novel host factor for the modulation of VACV egress and dissemination. Here, we demonstrate that (i) ISG15 regulates EV production and the formation of comet-shaped plaques, (ii) ISG15 regulates actin tail formation, (iii) ISG15 impacts the viral proteome composition, and (iv) ISG15 interacts with several VACV proteins (e.g., A36), although the outcome of these interactions remains to be elucidated. Altogether, our work highlights an essential role for ISG15/ISGylation in viral infection and cell-to-cell spread and points out the relevance of the comprehension of ISG15-mediated antiviral responses, which might lead to the development of effective therapies against relevant human pathogens.

## MATERIALS AND METHODS

### Cells and viruses.

Immortalized *Isg15^+/+^* and *Isg15^−/−^* MEFs (kindly provided by K. P. Knobeloch) (74) and NIH/3T3 (ATCC CRL-1658) cells were cultured in Dulbecco modified Eagle medium (DMEM) supplemented with 10% fetal calf serum (FCS). BSC40 (ATCC CRL-2761) cells were cultured in DMEM supplemented with 5% FCS. The FCS used in all experiments was heat inactivated (56°C, 30 min) prior to use. NIH 3T3 cells were transduced with lentiviral particles containing specific shRNAs for ISG15, yielding 3T3-L1 and 3T3-L2, or a scramble shRNA, yielding 3T3-SC. To generate viral stocks, the International Health Department-J (IHD-J) strain of vaccinia virus (VACV; kindly provided by Dolores Rodriguez), the IHD-J-ISG15 recombinant virus, and the Western Reserve (WR) VACV strain were grown in BSC40 cells (0.01 PFU/cell; 48 h) and purified by centrifugation through a sucrose cushion, followed by a sucrose gradient, as previously described (75). Virus aliquots were stored at −80°C.

### Virus titration.

Viral stocks, as well as samples of intracellular or extracellular virus, were titrated by plaque assay, as previously described (76), with slight modifications: DMEM–2% FCS containing 1% low-temperature-melting agarose was used as overlay medium for the IHD-J strain. Intracellular virus samples were subjected to three cycles of freeze-thawing to allow for the cell lysis. Extracellular virus samples were titrated freshly after centrifugation for 2 min at 250 × *g*.

### Comet-shaped plaque formation assays.

Cell monolayers in six-well plates were infected with ~0.0001 PFU/cell of IHD-J or WR per well in DMEM. After 1 h of incubation at 37°C and 5% CO_2_, cells were washed and incubated with DMEM supplemented with 2% FCS for an additional 48 (for IHD-J) or 72 h (for WR). Plaques were visualized after fixation and staining with 0.1% crystal violet in 10% formaldehyde in phosphate-buffered saline (PBS).

### Immunofluorescence.

Immortalized *Isg15^+/+^* or *Isg15^−/−^* MEFs were grown on 12-mm-diameter glass coverslips in DMEM–10% FCS to a confluence of 40 to 50% and mock infected or infected at a multiplicity of infection (MOI) of 2 PFU/cell with IHD-J. At the indicated times postinfection, cells were washed with PBS, fixed with 4% paraformaldehyde, permeabilized with 0.25% Triton X-100 in PBS for 30 min, and blocked in PBS with 10% FCS for 45 min at room temperature. Specific monoclonal antibodies for the VACV A27 protein (1:1,000; kindly provided by Mariano Esteban) and F13 protein (1:500; kindly provided by Rafael Blasco) were used as primary antibodies. Alexa Fluor 488-conjugated mouse (for A27) or Alexa Fluor 594-conjugated rat (for F13) IgG antibodies (Invitrogen) were used as secondary antibodies. Cell nuclei were stained with 4′,6′-diamidino-2-phenylindole (DAPI; Sigma, 1:200). F-actin was stained with rhodamine-conjugated phalloidin (Molecular Probes). Confocal microscopy was performed using a Leica SP8 laser scanning microscope, and images were collected and processed with LAS X software (Leica, Wetzlar, Germany).

### Electron microscopy.

Monolayers of *Isg15^+/+^* or *Isg15^−/−^* immortalized MEFs were infected at an MOI of 2 PFU/cell with the strain IHD-J of VACV. At 9 hpi, when the cytopathic effect was evident, the supernatant was removed, and the cells were fixed with a solution of 2.5% glutaraldehyde containing 1% tannic acid–0.4 M HEPES in PBS. After fixation, cells were carefully scraped, centrifuged to eliminate the fixative, and processed for embedding in the epoxy-resin EML-812 as previously described (77). Electron micrographs were taken using a transmission electron microscope (JEOL JEM-1011; Centro Nacional de Biotecnología, Madrid, Spain) equipped with a ES1000W Erlangshen charge-coupled-device camera (Gatan, Inc.) at an acceleration voltage of 40 to 100 kV. Fifty low-magnification micrographs were analyzed per genotype. Cells that could be visualized for integrity were considered for the analysis. Noninfected cells were excluded from the analysis. The number of intracellular viral particles per cell was quantified, with more than 110 cells analyzed per genotype.

### Protein analysis by Western blotting.

Immortalized *Isg15^+/+^* or *Isg15^−/−^* MEFs, NIH 3T3 cells, and lentivirus-transduced NIH 3T3 cells, or purified virions were collected, and lysates were obtained by solubilizing cells in SDS-Laemmli sample buffer supplemented with 100 mM dithiothreitol (DTT). Cell lysates were boiled for 5 min, resolved by sodium dodecyl sulfate-polyacrylamide gel electrophoresis (SDS-PAGE) with Laemmli running buffer and transferred to polyvinylidene difluoride membranes (Merck-Millipore) in a Trans-Blot SD Semi-Dry Transfer Cell (Bio-Rad) according to the manufacturer’s recommendations. Membranes were blocked with 5% skim milk in PBS containing 0.1% Tween 20 (PBS-T) and incubated with the corresponding primary antibodies, as indicated in the figure legends (actin and eif2α were from Santa Cruz, A27 was kindly provided by Mariano Esteban; F13 was kindly provided by Rafael Blasco, and A26 was kindly provided by Wen Chang) in 0.5% skim milk in PBS-T. Membranes were then washed with PBS-T and incubated with anti-rabbit or anti-mouse peroxidase-labeled antibodies (1:10,000; Sigma-Aldrich). After extensive washing with PBS-T, the immune complexes were detected by using Clarity Western ECL blot substrate (Bio-Rad) and a ChemiDoc XRS^+^ System (Bio-Rad), according to the manufacturer’s instructions. Protein quantity was analyzed using Fiji software (78).

### Generation of recombinant IHD-J-ISG15.

Monolayers of BSC-40 cells were infected with parental IHD-J at an MOI of 0.01 PFU/cell and transfected 1 h later with 10 μg of DNA of plasmid pCyA-ISG15GG (pCyA was kindly provided by Mariano Esteban and is described in reference 79) using LT-1 (Mirus Bio) according to the manufacturer’s recommendations. The gene ISG15GG was fused to a V5 tag sequence at its N-terminal domain to facilitate its detection. At 48 hpi, the cells were harvested, lysed by freeze-thaw cycling, sonicated, and used for recombinant-virus screening. Recombinant IHD-J containing the 513-bp DNA fragment encoding the ISG15-V5 fusion protein and transiently coexpressing the β-Gal reporter gene (IHD-J-ISG15, X-Gal^+^) were selected by three consecutive rounds of plaque purification in Neutral Red (Sigma, catalog no. N2889)- and X-Gal (5-bromo-4-chloro-3-indolyl-β-d-galactopyranoside; 40 mg/mL)-stained BSC-40 cells. In the following purification steps, recombinant IHD-J containing the ISG15 gene and lacking the β-Gal encoding gene (deleted by homologous recombination between the TK left arm and the short TK left arm repeat flanking the reporter gene) (IHD-J-ISG15, X-Gal^−^) were isolated by three additional consecutive rounds of plaque purification in X-Gal-stained (40 mg/mL) BSC40 cells, screening for nonstained viral plaques. In each round of purification, the isolated plaques were expanded in BSC-40 cells for 24 h, and the viruses obtained were used for the next purification round. To test the identity and purity of the recombinant virus IHD-J-ISG15, DNA was extracted from infected cells and subjected to PCR amplification using the oligonucleotides TK-L (5′-TGATTAGTTTGATGCGATTC-3′) and TK-R (5′-TGTCCTTGATACGGCAG-3′), with annealing in the TK flanking sequences. The resulting PCR product was analyzed by sequencing. ISG15 expression was analyzed by Western blotting with anti-V5 antibody (Invitrogen, catalog no. R960-25).

### Statistical analysis.

One-tailed or two-tailed unpaired Student *t* tests were used to analyze the differences in mean values between groups. All results are expressed as means ± the standard deviations (SD); *P* values of <0.05 were considered significant.

### LC-MS/MS of purified virions.

*Isg15^+/+^* or *Isg15^−/−^* MEFs were infected with IHD-J (MOI of 0.01, 48 h), scraped in culture medium, pelleted, and resuspended in 10 mM Tris (pH 9.0). Cells were disrupted by vigorous vortexing for 90 s. After sonication for 3 min, cell debris were pelleted at 1,000 × *g* and 4°C for 5 min. Purification of mature virions was done by rate-zonal sucrose gradient centrifugation in sterile SW28 centrifuge tubes (PMID 18265124). For this purpose, the supernatant was centrifuged through a 20% sucrose cushion at 32,900 × *g* and 4°C for 80 min, and the virus pellet was resuspended in 1 mL of 10 mM Tris (pH 9.0). The virus suspension was sonicated for 1 min, layered on a 20 to 45% continuous sucrose gradient, and centrifuged at 26,000 × *g* and 4°C for 50 min. The virus bands were pooled and concentrated at 32,900 × *g* and 4°C for 60 min. The pellet was resuspended in 1 mL of 10 mM Tris pH 9.0 and stored in aliquots at −80°C. Purified virions were processed for LC-MS/MS analysis.

### In-solution digestion.

Protein samples were individually digested with trypsin using a standard protocol. Briefly, 20 μg of protein of each sample were resuspended and denatured in 20 μL of 7 M urea–2 M thiourea–100 mM TEAB (triethylammonium bicarbonate), reduced with 2 μL of 50 mM Tris 2-carboxyethyl phosphine (TCEP; AB SCIEX, Foster City, CA; pH 8.0) at 37°C for 60 min, followed by cysteine-blocking reagent chloroacetamide (CAA). Samples were diluted up to 60 μL with 50 mM TEAB to reduce the concentration of urea. Then, 1 μg of sequence grade-modified trypsin (Pierce) was added to each sample (enzyme/sample ratio, 1:20), which was then incubated at 37°C overnight on a shaker. After digestion, the samples were dried in a SpeedVac (Thermo Scientific, Waltham, MA).

### Tagging with TMTsixplex reagent.

The resulting tryptic peptides were subsequently labeled using TMTsixplex Isobaric Mass Tagging kit (Thermo Scientific, Rockford, IL) according to the manufacturer’s instructions as follows: 126, WT-R1; 127, WT-R2; 128, WT-R3; 129, KO-R1; 130, KO-R2; and 131, KO-R3. After labeling, the samples were pooled, evaporated to dryness, and stored at −20°C until the LC-MS analysis. Three biological replicates of each condition were analyzed.

### LC-ESI-MS/MS.

Before MS analysis, we determined the amount of peptide in the combined sample by Qubit fluorometric quantitation (Thermo Fisher Scientific). A 1-μg aliquot of each fraction was subjected to 1D-nano LC-ESI-MS/MS (liquid chromatography electrospray ionization tandem mass spectrometry) analysis using an Ultimate 3000 nano HPLC system (Thermo Fisher Scientific) coupled online to an Orbitrap Exploris 240 mass spectrometer (Thermo Fisher Scientific). Peptides were eluted onto a 50-cm × 75-μm Easy-spray PepMap C_18_ analytical column at 45°C and were separated at a flow rate of 300 nL/min using a 120 min gradient ranging from 2 to 35% mobile phase B (mobile phase A, 0.1% formic acid [FA]; mobile phase B, 80% acetonitrile [ACN] in 0.1% FA). The loading solvent was 2% ACN in 0.1% FA, and the injection volume was 5 μL. Data acquisition was performed using a data-dependent top-20 method, in full scan positive mode, scanning 375 to 1,200 *m/z*. Survey scans were acquired at a resolution of 60,000 at *m/z* 200, with normalized automatic gain control (AGC) target (%) of 300 and a maximum injection time (IT) in AUTO. The top 20 most intense ions from each MS1 scan were selected and fragmented via higher-energy collisional dissociation (HCD). The resolution for HCD spectra was set to 45,000 at *m/z* 200, with an AGC target of 100 and a maximum ion injection time in AUTO. Isolation of precursors was performed with a window of 0.7 *m/z* and an exclusion duration of 45 s, and the HCD collision energy was 30. Precursor ions with single, unassigned, or six and higher charge states from fragmentation selection were excluded. Raw instrument files were converted to MGF files and MS/MS spectra searched using OMSSA 2.1.9, X! Tandem 2013.02.01.1, Myrimatch 2.2.140, and MS-GF+ (Beta v10072) against a composite target/decoy database built from the Mus musculus reference proteome sequences and the vaccinia virus (strain Western Reserve) proteome downloaded from UniProtKB. Search engines were configured to match potential peptide candidates with mass error tolerance of 25 ppm and fragment ion tolerance of 0.02 Da, allowing for up to two missed tryptic cleavage sites and a maximum isotope error (13C) of 1, considering fixed carbamidomethyl modification of cysteine and variable oxidation of methionine, pyroglutamic acid from glutamine or glutamic acid at the peptide N terminus, and modification of lysine and peptide N terminus with TMTsixplex reagents. Score distribution models were used to compute peptide-spectrum match *P* values, and spectra recovered by FDR < 0.01 (peptide-level) filter were selected for quantitative analysis. Approximately 5% of the signals with the lowest quality were removed prior to further analysis. Differential regulation was measured using linear models, and statistical significance was measured using *q* values (FDR). All analyses were conducted using software from Proteobotics (Madrid, Spain).

### Immunoprecipitation assay.

Sucrose gradient-purified intracellular virions of IHD-J and IHD-J-ISG15 strains, previously sonicated, were resuspended in a 50 mM Tris-HCl (pH 7.5) buffer, containing 150 mM NaCl and 1% NP-40 detergent and supplemented with a protease inhibitor cocktail mini-tablet (complete from Roche) and a phosphatase inhibitor mini-tablet (Pierce from Thermo Scientific) according to the manufacturer’s instructions. The virus extract was vortexed and incubated on ice for 15 min. The amount of protein in the samples was determined by the Bradford protein method. For immunoprecipitation, 70 μg of protein extract was incubated with the anti-V5 antibody (diluted 1:50) overnight at 4°C; then, protein G-Sepharose beads were added to the mixture, and the sample was further incubated for 2 h at 4°C. The beads were washed four times with a 50 mM Tris-HCl (pH 7.5) buffer containing 150 mM NaCl and 0.1% NP-40 detergent and finally washed once with PBS. The immunoprecipitated proteins were eluted with SDS sample buffer and analyzed by immunoblotting.

### Protein identification of immunoprecipitated extract.

Protein eluted from the immunoprecipitation assay (three biological replicates per condition) were individually loaded and concentrated in a 12% SDS-PAGE gel. Each sample was divided in four to five bands, and these were digested with trypsin using an automatic robot Opentrons. In all cases, digestion was performed according to a protocol described by Shevchenko et al. (80). In summary, gel plugs were washed with 50 mM ammonium bicarbonate and samples reduced with 10 mM DTT. Alkylation was carried out with 10 mM chloroacetamide at room temperature before adding recombinant sequencing-grade trypsin (0.1 μg, Promega). Digestion took place at 37°C for 18 h. Following digestion, peptides were extracted, pooled, dried by speed-vac centrifugation, and stored at −20°C until needed.

### LC-ESI-MS/MS analysis and database searching.

Nano LC ESI-MS/MS analysis was performed using a Thermo Ultimate 3000 nanoHPLC coupled to a Thermo Orbitrap Exploris 240 mass spectrometer (Thermo Fisher Scientific, San José, CA). The analytical column used was an Easy-spray PepMap RSLC C_18_ reversed-phase column (75 μm × 50 cm, 2-μm particle size). The trap column was an Acclaim PepMap 100 (5-μm particle diameter, 100 Å pore size) switched on-line with the analytical column. The loading pump delivered a solution of 0.1% formic acid in 98% water–2% acetonitrile (Merck, Germany) at 10 μL/min. The nanopump provided a flow rate of 250 nL/min and was operated under gradient elution conditions, using 0.1% formic acid (Fluka, Buchs, Switzerland) in water as mobile phase A and 0.1% formic acid in 80% acetonitrile as mobile phase B. Gradient elution was performed according the following scheme: isocratic conditions of 94% A–6% B for 2 min, a linear increase to 35% B in 40 min, a linear increase to 95% B in 1 min, isocratic conditions of 95% B for 4 min and return to initial conditions in 1 min. The injection volume was 5 μL. The LC system was coupled via an Easy-Spray nanospray source to the mass spectrometer. Automatic data-dependent acquisition using dynamic exclusion allowed obtaining both full scan (*m/z* 350 to 1,250) MS spectra, followed by tandem MS HCD spectra, of the 20 most abundant ions. MS and MS/MS data were used to search against a composed database containing *Chlorocebus sabaeus* and *Vaccinia virus* protein sequences (downloaded from UniProtKB, June 2021). The database also contained a short list of common laboratory contaminants downloaded from CRAP Database (https://reprint-apms.org/). Searches were done using a licensed version of Peaks v.7.5, and search parameters were set as follows: carbamidomethyl cysteine as fixed modification and acetyl (protein N-term), NQ deamidation, -GG at Lys residues, Glu to pyroglutamic and oxidized methionine as variable ones. Peptide mass tolerance was set at 10 ppm and 0.02 Da for MS and MS/MS spectra, respectively, and two missed cleavages were allowed. The FDR was set at 1% at the peptide level. Only proteins with at least one unique peptide were considered.
