# Supplementary material for: ISG15 Is Required for the Dissemination of Vaccinia Virus Extracellular Virions
Source: Microbiol Spectr. 2023 Apr 10;11(3):e04508-22. doi: 10.1128/spectrum.04508-22 (PMC10269806; doi:10.1128/spectrum.04508-22)
Supplement: Supplemental file 1 — Supplemental material. Download spectrum.04508-22-s0001.pdf, PDF file, 0.6 MB [file spectrum.04508-22-s0001.pdf]

Figure S1

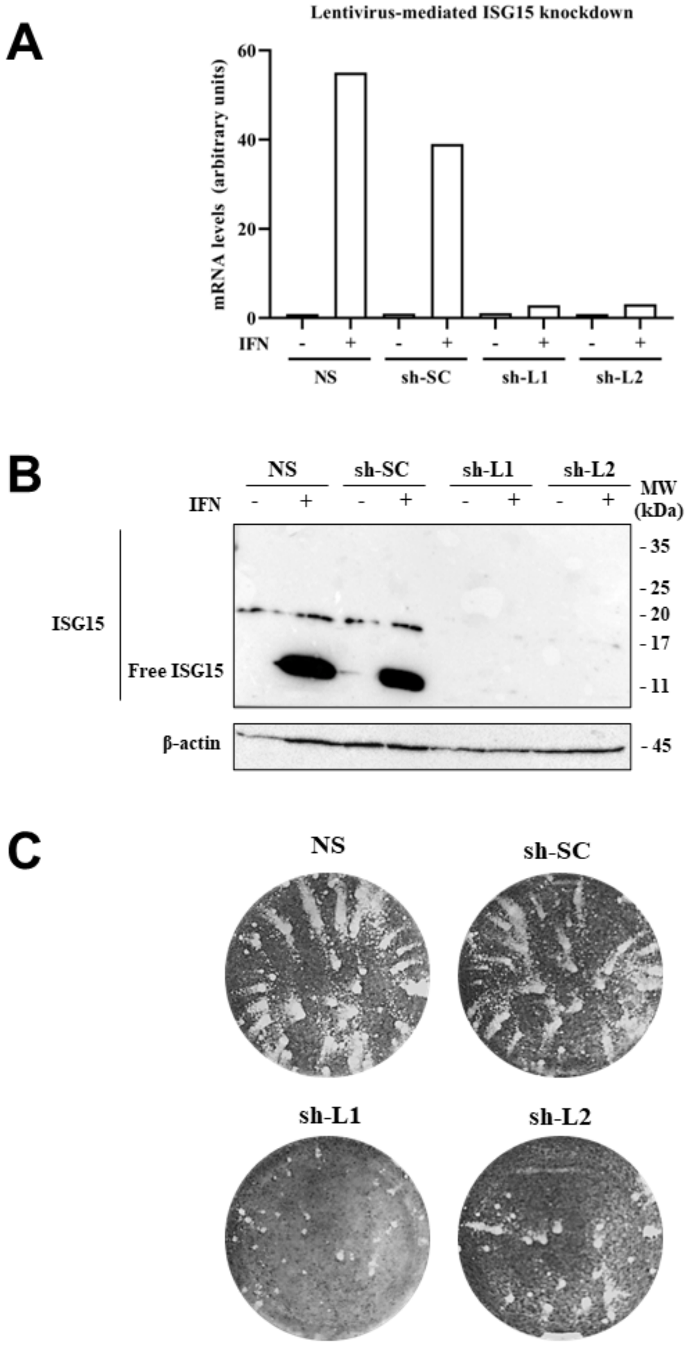

Supplementary Figure 1. Lentivirus-mediated knockdown of ISG15 in NIH-3T3 cells blocks the formation of comet-like plaques during IHD-J infection. To knockdown ISG15 expression, NIH-3T3 cells were non-transduced (NS) or transduced with lentivirus expressing ISG15-specific shRNAs (sh-L1 and sl-L2), or scrambled shRNA (sh-SC) used as a negative control. ISG15 inhibition was determined by RT-qPCR **(A)** and by Western Blot **(B)** in untreated cells (IFN -) or after treatment with 500 units/mL for 16 h (IFN +) Note that the low basal expression of ISG15 in these cells makes it almost undetectable in the absence of IFN treatment. **(C)** Monolayers of NIH-3T3, non transduced or transduced with lentivirus expressing sh-L1, sh-L2 or sh-SC, were infected with IHD-J (0.0001 PFU/cell) for 48 h, and comet-like plaques were visualized by staining with 0.1% crystal violet in 10% formaldehyde in PBS.

Figure S2

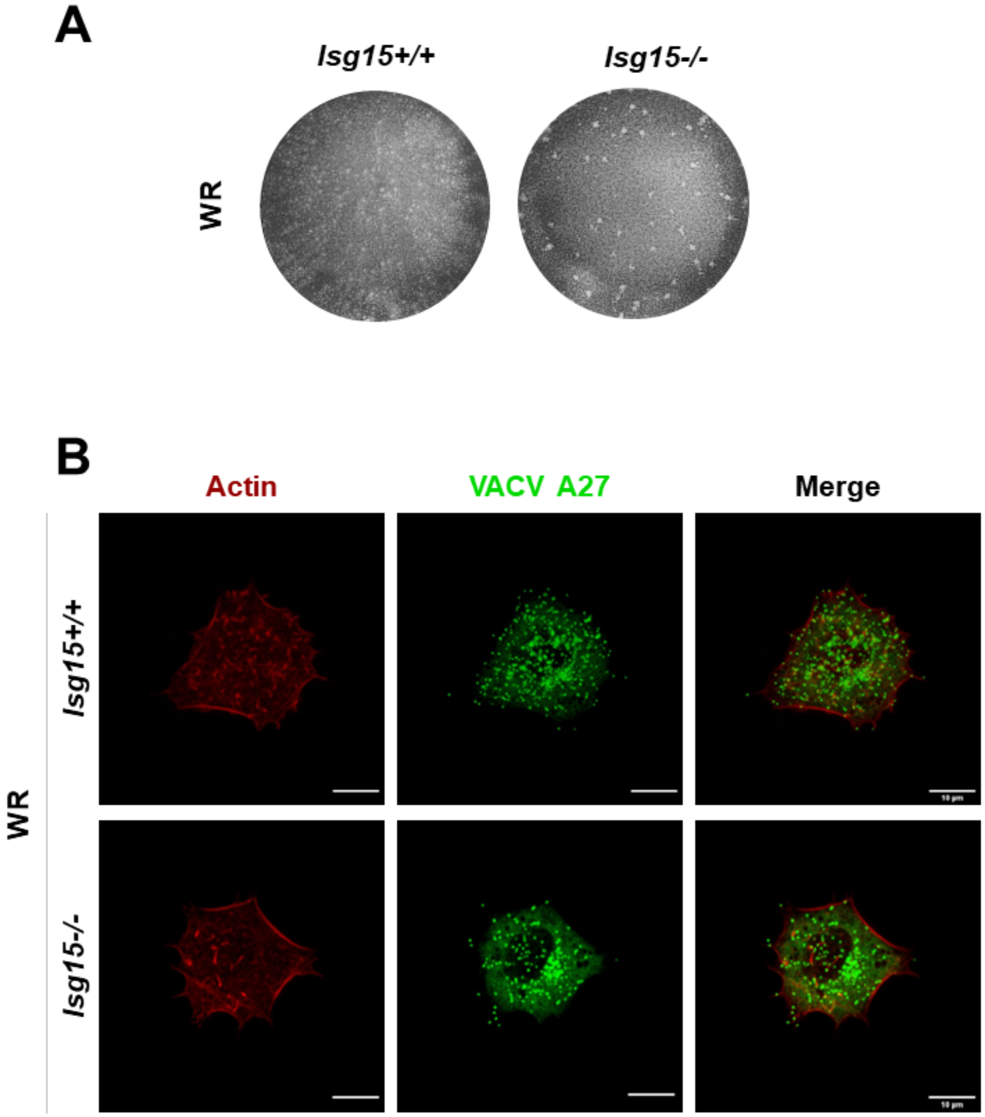

Supplementary Figure 2. Evaluation of EV release, actin tail and comet-like plaque formation during infection with VACV WR strain. (A) WR releases less extracellular virus than IHD-J. Immortalized *Isg15+/+* and *Isg15-/-* MEFs were infected with IHD-J or WR (PFU 0.01/cell) and virus secreted to the supernatant was determined at 48 h post-infection **(B) The formation of comet-like plaques is impaired in WR-infected *Isg15-/-* MEFs.** Monolayers of immortalized ISG15$^{+/+}$ or ISG15$^{-/-}$ MEFs were infected with WR for 48 h and comet-like plaques (liquid medium) were visualized by staining with 0.2% crystal violet in 10% formaldehyde in PBS. **(C) The formation of actin tails is reduced in WR-infected *Isg15-/-* MEFs.** Immortalized ISG15$^{+/+}$ or ISG15$^{-/-}$ MEFs growing in 12-mm coverslips were infected with WR at 0.5 PFU/cell. At 9 hpi, actin tails were visualized by phalloidin-staining (red), and viral particles were visualized using an anti-A27 antibody, followed by a fluorescent secondary antibody (green).

Figure S3

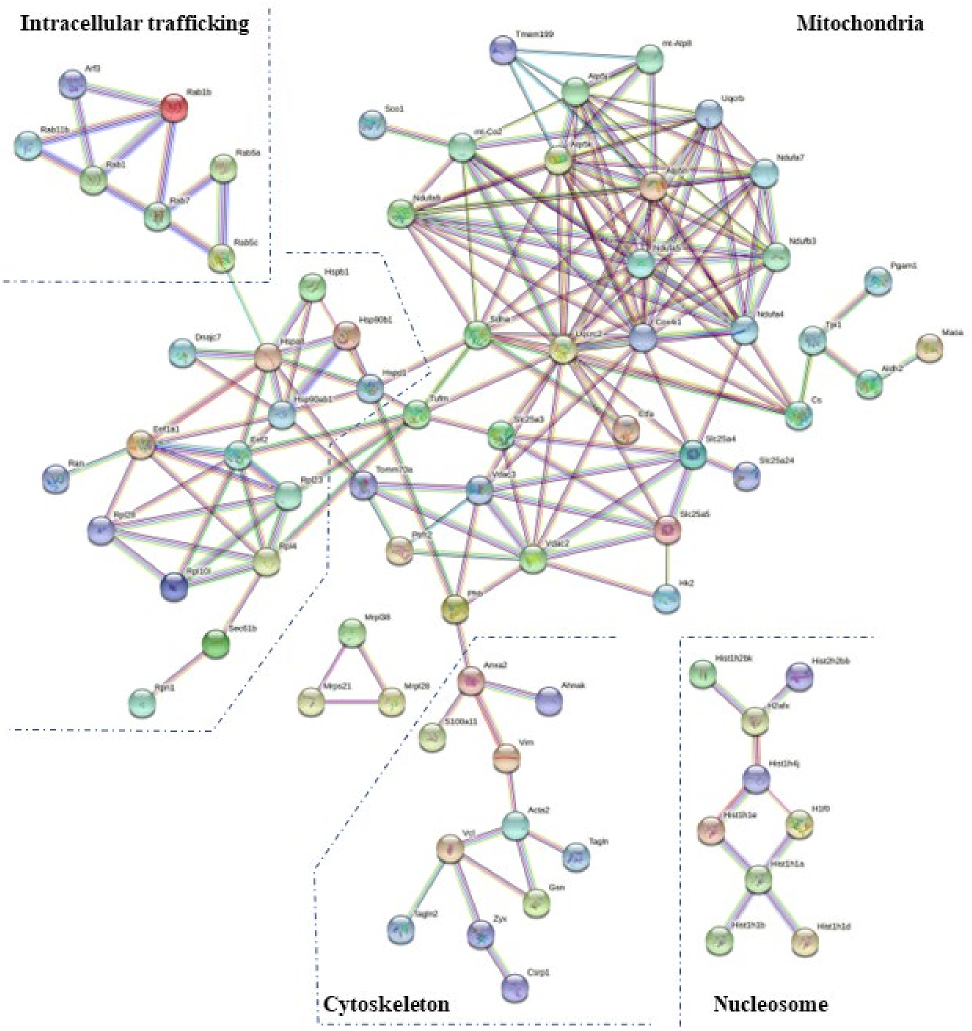

Supplementary Figure 3. Cellular proteins identified in sucrose gradient-purified IHD-J virions from *Isg15-/-* and *Isg15+/+* MEFs. STRING Database V11.5 was used to generate protein-protein interaction networks and to classify host proteins identified in the proteomic analysis of purified virions that were significantly increased or decreased (*P-value* ≤ 0.01) in virions from *Isg15-/-* cells, compared to their *Isg15+/+* counterparts. Functional and physical protein associations were analyzed, and the minimum required interaction score was set to 0.9 (highest confidence).
